# Sozioökonomische Deprivation und vorzeitige Sterblichkeit in Deutschland 1998–2021: Eine ökologische Studie mit What-if-Szenarien der Ungleichheitsreduktion

**DOI:** 10.1007/s00103-024-03862-0

**Published:** 2024-04-08

**Authors:** Jens Hoebel, Enno Nowossadeck, Niels Michalski, Jens Baumert, Benjamin Wachtler, Fabian Tetzlaff

**Affiliations:** 1 Fachgebiet Soziale Determinanten der Gesundheit, Abteilung für Epidemiologie und Gesundheitsmonitoring, Robert Koch-Institut, Berlin, Deutschland; 2 Fachgebiet Körperliche Gesundheit, Abteilung für Epidemiologie und Gesundheitsmonitoring, Robert Koch-Institut, Berlin, Deutschland

**Keywords:** Soziale Determinanten, Gesundheitliche Ungleichheit, Sozioökonomische Faktoren, Vorzeitige Sterblichkeit, Regionale Deprivation, Social determinants, Health inequalities, Socioeconomic factors, Premature mortality, Area deprivation

## Abstract

**Hintergrund:**

Das frühere Versterben in sozioökonomisch benachteiligten Bevölkerungsgruppen stellt eine extreme Ausprägungsform gesundheitlicher Ungleichheit dar. Diese Studie untersucht das Ausmaß, die zeitliche Entwicklung und Reduktionspotenziale regionaler sozioökonomischer Ungleichheiten in der vorzeitigen Sterblichkeit in Deutschland.

**Methoden:**

Bundesweite Daten der amtlichen Todesursachenstatistik wurden auf Stadt- und Landkreisebene mit amtlichen Bevölkerungsdaten und dem „German Index of Socioeconomic Deprivation“ (GISD) verknüpft. Altersstandardisierte Mortalitätsraten für unter 75-Jährige wurden stratifiziert nach Geschlecht und Deprivationsquintil berechnet. In einer What-if-Analyse wurde anhand kontrafaktischer Szenarien berechnet, wie viel niedriger die vorzeitige Sterblichkeit insgesamt läge, wenn sozioökonomische Mortalitätsungleichheiten verringert würden.

**Ergebnisse:**

Männer und Frauen im höchsten Deprivationsquintil hatten ein 43 % bzw. 33 % höheres Risiko, vorzeitig zu versterben, als Gleichaltrige im niedrigsten Deprivationsquintil. Höhere Mortalitätsraten mit steigender Deprivation zeigten sich für die Herz-Kreislauf- und Krebsmortalität, aber auch für andere Todesursachen. Die sozioökonomischen Mortalitätsungleichheiten nahmen bereits vor der COVID-19-Pandemie zu und verschärften sich in den ersten Jahren der Pandemie weiter. Hätten alle Regionen die gleiche Mortalität wie jene im niedrigsten Deprivationsquintil, läge die vorzeitige Sterblichkeit insgesamt 13 % niedriger.

**Diskussion:**

Die zunehmende Ungleichheit in der vorzeitigen Sterblichkeit zwischen deprivierten und wohlhabenden Regionen verdeutlicht, dass die Herstellung gleichwertiger Lebensverhältnisse im Bundesgebiet auch für die Verringerung der gesundheitlichen Ungleichheit ein wichtiges Handlungsfeld darstellt.

**Zusatzmaterial online:**

Zusätzliche Informationen sind in der Online-Version dieses Artikels (10.1007/s00103-024-03862-0) enthalten.

## Abstract (en)

**Background:**

Earlier mortality in socioeconomically disadvantaged population groups represents an extreme manifestation of health inequity. This study examines the extent, time trends, and mitigation potentials of area-level socioeconomic inequalities in premature mortality in Germany.

**Methods:**

Nationwide data from official cause-of-death statistics were linked at the district level with official population data and the German Index of Socioeconomic Deprivation (GISD). Age-standardized mortality rates before the age of 75 were calculated stratified by sex and deprivation quintile. A what-if analysis with counterfactual scenarios was applied to calculate how much lower premature mortality would be overall if socioeconomic mortality inequalities were reduced.

**Results:**

Men and women in the highest deprivation quintile had a 43% and 33% higher risk of premature death, respectively, than those in the lowest deprivation quintile of the same age. Higher mortality rates with increasing deprivation were found for cardiovascular and cancer mortality, but also for other causes of death. Socioeconomic mortality inequalities had started to increase before the COVID-19 pandemic and further exacerbated in the first years of the pandemic. If all regions had the same mortality rate as those in the lowest deprivation quintile, premature mortality would be 13% lower overall.

**Discussion:**

The widening gap in premature mortality between deprived and affluent regions emphasizes that creating equivalent living conditions across Germany is also an important field of action for reducing health inequity.

## Hintergrund

Eine Vielzahl sozialepidemiologischer Untersuchungen belegt den engen Zusammenhang zwischen der sozioökonomischen und der gesundheitlichen Lage [1–6]. Die Befunde zeigen mit großer Übereinstimmung, dass Menschen in sozioökonomisch benachteiligten Verhältnissen verminderte Gesundheitschancen und erhöhte Krankheitsrisken aufweisen. Daneben können gesundheitliche Beeinträchtigungen auch zu einer Verschlechterung der sozioökonomischen Lage führen [7]. Diese gesundheitliche Ungleichheit spiegelt sich letztlich in einer früheren Sterblichkeit und kürzeren Lebenserwartung in sozioökonomisch benachteiligten gegenüber vergleichsweise privilegierten Gruppen wider [8–10], was als eine extreme Ausprägungsform der gesundheitlichen Ungleichheit erachtet wird [11].

Das durchschnittlich frühere Versterben in sozioökonomisch benachteiligten Bevölkerungsgruppen ist auch in einem wohlhabenden Land wie Deutschland trotz breit ausgebauter Sozial- und Versorgungssysteme deutlich zu beobachten [12, 13]. Die Möglichkeiten zur Untersuchung von Art und Ausmaß sozioökonomischer Ungleichheiten in der Mortalität sind in Deutschland jedoch erheblich eingeschränkter als in vielen anderen Hocheinkommensländern, weil hierzulande keine nationalen Sterbedaten mit sozioökonomischen Informationen vorhanden sind. Dies ist vor allem den Umständen geschuldet, dass im deutschen Zensus kein Mortalitäts-Follow-up durchgeführt wird, dass auf den Todesbescheinigungen keine sozioökonomischen Merkmale der Verstorbenen erfasst werden und dass kein bundesweites Mortalitätsregister existiert, welches mit Sozialdaten verknüpft werden könnte [14, 15]. Somit müssen hierzulande andere Daten und Methoden für entsprechende Analysen herangezogen werden. Neben Langzeitdaten auf Basis von Bevölkerungsstichproben [12, 13] und Routinedaten der Sozialversicherungen [16–18] ermöglichen ökologische Studiendesigns, diese Datenlücke in Deutschland zu überbrücken. Dabei werden die in amtlichen Sterbedaten verfügbaren Informationen zum Wohnort der Verstorbenen genutzt, um diese mit regionalen Sozialdaten zu verknüpfen [19–22].

Über die letzten Jahrzehnte ist die vorzeitige Sterblichkeit in vielen Ländern Europas deutlich gesunken [23, 24]. Neben Verbesserungen und Innovationen im Bereich der medizinischen Versorgung und Prävention, z. B. von Herz-Kreislauf- und Krebserkrankungen, dürften auch Verbesserungen der Lebens- und Arbeitsbedingungen zu dieser Entwicklung beigetragen haben. Trotz dieser Gesamtentwicklung besteht die stark ausgeprägte Ungleichheit im Sterbegeschehen zuungunsten von Bevölkerungsgruppen in sozioökonomisch benachteiligten Verhältnissen innerhalb europäischer Gesellschaften weiter fort [10, 23, 25, 26]. In Deutschland lässt sich diese Ungleichheit auch regional erkennen. So werden hierzulande beträchtliche Unterschiede in der Mortalität und Lebenserwartung zwischen den Regionen beobachtet, die sich zu großen Anteilen durch sozioökonomische Faktoren erklären lassen [19, 27–29]. Somit wird die vorzeitige Sterblichkeit bzw. ihre räumliche Ungleichverteilung in dieser Debatte auch als aussagekräftiger Indikator für die zu erreichende Gleichwertigkeit der Lebensverhältnisse im Bundesgebiet gemäß Artikel 72 des deutschen Grundgesetzes erachtet [28]. Betrachtungen zeitlicher Trends sowie todesursachenspezifischer Muster liegen diesbezüglich für Deutschland bislang jedoch kaum vor.

Die Verbesserung gesundheitlicher Chancengleichheit und die Verringerung gesundheitlicher Ungleichheit sind erklärte Kernziele von Public Health und Teil der von der Weltgesundheitsorganisation (WHO) festgelegten „Essential Public Health Operations“ [30, 31]. In der Public-Health-Forschung und Epidemiologie fehlt es jedoch häufig an empirischen Untersuchungen zu der Frage, wie groß die Potenziale einer Reduktion der gesundheitlichen Ungleichheit für die Gesundheit der Bevölkerung insgesamt sind. Eines der noch wenigen Beispiele für solche Berechnungen liefert eine Studie aus dem Vereinigten Königreich. Sie zeigt, dass knapp 36 % der vorzeitigen Sterblichkeit in der Bevölkerung Englands auf die regionale sozioökonomische Ungleichheit zurückzuführen („attributable“) sind [25]. Dies bedeutet in Bezug auf die oben genannte Frage, dass mehr als ein Drittel der gesamten vorzeitigen Sterblichkeit in England durch die nahezu vollständige Eliminierung der regionalen sozioökonomischen Mortalitätsungleichheit, hier angenommen durch eine Senkung der vorzeitigen Sterblichkeit in deprivierten Regionen auf das Niveau in wohlhabenden Regionen, vermieden werden könnte. Würden solche Quantifizierungen systematisch und für verschiedene Maße der Bevölkerungsgesundheit durchgeführt, könnte dies einen Beitrag dazu leisten, unterschiedliche Strategien zur Verminderung der gesundheitlichen Ungleichheit abzuwägen und dadurch besser priorisieren zu können. Zudem könnten sie Informationen für politische Entscheidungsprozesse bereitstellen, den politischen Willen zur Verringerung der gesundheitlichen Ungleichheit stärken und dazu beitragen, Investitionen in entsprechende Maßnahmen zu rechtfertigen [6, 32]. Für Deutschland stehen epidemiologische Untersuchungen aus diesem Blickwinkel bislang noch weitgehend aus.

In diesem Artikel wird den Fragen nachgegangen, (1) wie stark die regionalen sozioökonomischen Ungleichheiten in der vorzeitigen Sterblichkeit in Deutschland ausgeprägt sind, (2) ob sich diese Ungleichheiten über die letzten 2 Jahrzehnte in Art und Ausmaß verändert haben und (3) wie stark sich die vorzeitige Sterblichkeit in Deutschland insgesamt reduzieren ließe, wenn die sozioökonomischen Mortalitätsungleichheiten zwischen den Regionen des Landes verringert würden. Dabei werden neben Betrachtungen der vorzeitigen Gesamtsterblichkeit auch ursachenspezifische Betrachtungen für die beiden häufigsten Gruppen von Todesursachen, die Herz-Kreislauf- und Krebserkrankungen, angestellt.

## Methoden

Für die vorliegende Studie wurden Daten der amtlichen Todesursachenstatistik [33], der amtlichen Fortschreibung des Bevölkerungsstandes und des „German Index of Socioeconomic Deprivation“ (GISD) für die Jahre 1998 bis 2021 auf kleinräumiger Ebene miteinander verknüpft (ökologisches Studiendesign). Die amtliche Todesursachenstatistik ist eine bundesweite Vollerhebung aller Sterbefälle mit amtlicher Todesbescheinigung und Wohnsitz in Deutschland. Das für den Sterbefall zuständige Gesundheitsamt übermittelt die vorgesehenen Teile der Todesbescheinigung an das jeweilige Statistische Landesamt. Mithilfe der 10. Revision der „International Statistical Classification of Diseases and Related Health Problems“ (ICD-10) wird das sogenannte Grundleiden ermittelt, das als ursächlich für den Tod angesehen wird. Neben dem Geburts- und Sterbedatum, dem Geschlecht und der Todesursache liegen auch Informationen zum Wohnort der Verstorbenen vor. Dadurch können die Daten unter Beachtung datenschutzrechtlicher Bestimmungen und Geheimhaltungsregeln kleinräumig ausgewertet und mit anderen Regionaldaten verknüpft werden. Um die in der Todesursachenstatistik erfassten Sterbefälle auf die jeweilige Bevölkerung des Wohnorts beziehen zu können, wurde die Todesursachenstatistik mit kleinräumigen Daten der amtlichen Fortschreibung des Bevölkerungsstandes verknüpft. Die Verknüpfung der Daten erfolgte auf Ebene der 400 Landkreise und kreisfreien Städte, jeweils in 5‑Jahres-Altersgruppen innerhalb der Kreise sowie getrennt für Männer und Frauen, um altersstandardisierte und geschlechtsspezifische Analysen durchführen zu können.

### Vorzeitige Sterblichkeit

Die vorzeitige Sterblichkeit wurde in der vorliegenden Studie als „Versterben vor einem Alter von 75 Jahren“ definiert. Die Altersgrenze von unter 75 Jahren entspricht dem Grenzwert zur Definition vorzeitiger Sterblichkeit, wie er derzeit von der Organisation für wirtschaftliche Zusammenarbeit und Entwicklung (OECD) und Eurostat, dem statistischen Amt der Europäischen Union (EU), für die Berechnung vermeidbarer Sterblichkeit verwendet wird. Dabei orientiert sich dieser Grenzwert an den Ländern in der OECD und EU mit der kürzesten Lebenserwartung bei Geburt [34]. Für die in diesem Artikel dargestellten Analysen wurde sowohl die vorzeitige Gesamtsterblichkeit als auch die ursachenspezifische Sterblichkeit betrachtet. Differenziert wurde dabei zwischen den beiden häufigsten Todesursachen, den Herz-Kreislauf- (Todesursachen: ICD I00–I99) und Krebserkrankungen (Todesursachen: ICD C00–C97, ohne C44 und C77–C79), sowie der Gruppe anderer Todesursachen (restliche ICD-Codes). Sterbefälle mit ungültigen Todesursachencodes (sinkender Anteil über die Zeit [35], hauptsächlich ICD-Codes in Kapitel R [36]) wurden alters- und geschlechtsspezifisch für jedes Jahr und jeden Kreis proportional auf alle gültigen Todesursachencodes umverteilt.

### Regionale sozioökonomische Deprivation

Da die Todesursachenstatistik selbst keine Informationen zum sozioökonomischen Status der Verstorbenen enthält, wurden sozioökonomische Regionaldaten zum Wohnort der Verstorbenen mit den Todesursachenstatistik-Daten verknüpft, um den sozioökonomischen Status kleinräumig zu approximieren. Dafür wurde der German Index of Socioeconomic Deprivation (GISD; [19, 37]) jahresweise auf Ebene der Landkreise und kreisfreien Städte an die Sterbe- und Bevölkerungsdaten angespielt. Der GISD ist ein Maß relativer sozioökonomischer Deprivation der Regionen in Deutschland und beruht auf 9 räumlich aggregierten Einzelindikatoren zur Abbildung der 3 Kerndimensionen des soziökonomischen Status (Bildung, Beschäftigung, Einkommen). Jede Dimension wird mit jeweils 3 Einzelindikatoren repräsentiert. Beispiele für diese sind der Anteil von Schulabgängerinnen und Schulabgängern ohne Abschluss, die Arbeitslosenquote oder das durchschnittliche Haushaltsnettoeinkommen in den Regionen. Die Einzelindikatoren wurden in ihrer Dimension anhand ihrer Faktorladungen gewichtet, die mittels Hauptkomponentenanalyse ermittelt wurden. Anschließend gingen die 3 Dimensionen Bildung, Beschäftigung und Einkommen gleichgewichtet in den Gesamtindex ein, der einen Wert zwischen 0 (niedrigste Deprivation) und 1 (höchste Deprivation) annehmen kann. Für stratifizierte Analysen wurde der Index in Quintile eingeteilt, von Quintil 1 (niedrige Deprivation) bis Quintil 5 (hohe Deprivation). Weitere Details zum GISD, z. B. zu den zugrunde liegenden Daten sowie zur Gewichtung und zu Korrelationen der Einzeldimensionen, finden sich bei Michalski et al. [19].

### Statistische Analyse

Als Maßzahl für die vorzeitige Sterblichkeit wurde die standardisierte Mortalitätsrate als Anzahl der Todesfälle pro 100.000 Personen im Alter unter 75 Jahren mit Stratifizierung nach Geschlecht und Deprivationsquintil berechnet. Die Standardisierung erfolgte per direkter Altersstandardisierung auf die Europastandardbevölkerung 2013 [38]. Als Maß des relativen Risikos wurde das standardisierte Mortalitätsratenverhältnis (Standardized Mortality Ratio [SMR]) zwischen den Deprivationsquintilen berechnet.

Die Berechnungen erfolgten für jedes Kalenderjahr im Beobachtungszeitraum. Zunächst werden Ergebnisse für das Jahr 2019, dem letzten Jahr vor Beginn der COVID-19-Pandemie, dargestellt, um Einflüsse der Pandemie in dieser Betrachtung auszuschließen und eine Bestandsaufnahme zum Ausmaß regionaler sozioökonomischer Unterschiede in der vorzeitigen Sterblichkeit vor der Pandemie zu erstellen. Um zeitliche Entwicklungen und Trends zu erkennen, werden anschließend Verläufe der jährlichen Mortalitätsraten über den gesamten Beobachtungszeitraum von 1998 bis 2021 dargestellt. Zur Identifikation von Perioden mit ähnlichen jährlichen Mortalitätsveränderungen, wurden Joinpoint-Regressionen mit modifiziertem Bayes’schen Informationskriterium (BIC) stratifiziert nach Geschlecht und Deprivation berechnet [39, 40].

Um zu quantifizieren, in welchem Maß die vorzeitige Sterblichkeit insgesamt reduziert werden könnte, wenn die regionale sozioökonomische Ungleichheit in der Mortalität verringert würde, wurde eine What-if-Analyse mit verschiedenen kontrafaktischen Szenarien durchgeführt. Diese nehmen die Perspektive einer „Nivellierung nach oben“ („levelling up“ approach [32, 41]) ein, also einer Angleichung der Gesundheits- und Lebenschancen niedriger sozioökonomischer Gruppen auf das jeweilige Niveau höherer sozioökonomischer Gruppen:

*Szenario 1:* Menschen in allen Regionen Deutschlands haben das gleiche Risiko, vorzeitig zu versterben, wie jene in den sozioökonomisch am besten gestellten Regionen (Angleichung der Mortalität in den Deprivationsquintilen 2 bis 5 an die Mortalität in Deprivationsquintil 1).

*Szenario 2:* Das Risiko für vorzeitige Sterblichkeit in höher deprivierten Regionen wird auf das Niveau in den sozioökonomisch nächst bessergestellten Regionen gesenkt (Angleichung der Mortalität der Deprivationsquintile 2 bis 5 an das nächst weniger deprivierte Quintil).

*Szenario 3:* Das Risiko für vorzeitige Sterblichkeit in Regionen mit überdurchschnittlicher Deprivation verbessert sich auf das Niveau in Regionen mit mittlerer Deprivation (Angleichung der Mortalität in den Deprivationsquintilen 4 und 5 an die Mortalität in Deprivationsquintil 3).

Für die What-if-Analyse wurde zunächst die Anzahl der im jeweiligen Szenario erwarteten Todesfälle berechnet, indem die Mortalitätsrate der Bevölkerung in den niedrigeren Deprivationsquintilen (z. B. in Szenario 1 die Mortalitätsrate in Deprivationsquintil 1) auf die Bevölkerung in den höheren Deprivationsquintilen (z. B. in Szenario 1 der Deprivationsquintile 2 bis 5) stratifiziert nach 5‑Jahres-Altersgruppen, Geschlecht und Jahr angewendet wurde [25]. Durch Aufsummieren der Sterbefallzahlen über die Altersgruppen wurden die Gesamtzahlen der in jedem Deprivationsquintil beobachteten und erwarteten vorzeitigen Sterbefälle berechnet. Anschließend wurden diese jeweils über alle Deprivationsquintile aufsummiert, um die Differenz zwischen den beobachteten und erwarteten Sterbefällen insgesamt zu bilden. Diese Differenz gibt die Anzahl der vorzeitigen Sterbefälle an, die reduziert werden könnte, wenn die regionale sozioökonomische Ungleichheit in der Mortalität gemäß dem jeweiligen Szenario verringert würde. Zusätzlich wurde der prozentuale Anteil der im Szenario reduzierten Sterbefälle an allen beobachteten Sterbefällen berechnet, um zu ermitteln, um wie viel Prozent die vorzeitige Sterblichkeit im jeweiligen Szenario insgesamt reduziert wäre. Dieser Ansatz ist eng verbunden mit dem der „Population Attributable Fraction“ [42], welcher in der Epidemiologie angewendet wird, um die mit bestimmten Risikofaktoren verbundene Krankheitslast oder Sterblichkeit in Bevölkerungen zu quantifizieren. Dies gilt insbesondere für Szenario 1, das eine Nivellierung der Mortalität in der Gesamtbevölkerung an das Niveau in der sozioökonomisch am besten gestellten Bevölkerungsgruppe annimmt, wie es auch bei der Berechnung der vorzeitigen Sterblichkeit, die der sozioökonomischen Ungleichheit zurechenbar ist („ungleichheitsattributable Sterblichkeit“), erfolgt [25].

Die Analysen mit Einbeziehung von Mikrodaten der Todesursachenstatistik wurden an Gastwissenschaftsarbeitsplätzen (GWAP) des Amts für Statistik Berlin-Brandenburg mithilfe der Statistiksoftware Stata SE 17.0 (StataCorp LLC, College Station, TX, USA) durchgeführt. Dort aggregierte Daten wurden anschließend am Robert Koch-Institut (RKI) mit Stata und dem Joinpoint Regression Program (4.6.0.0, April 2018; Statistical Research and Applications Branch, National Cancer Institute, Bethesda, MD, USA) [39] weiter analysiert.

## Ergebnisse

**Figure 1:**
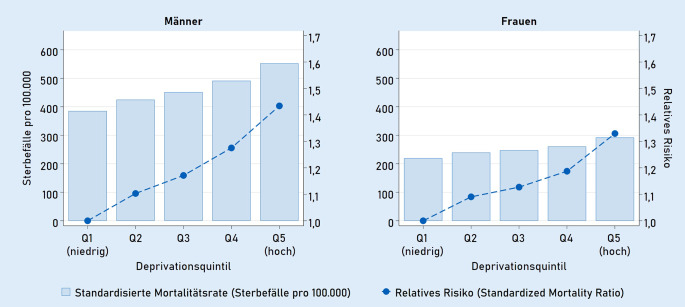

Über den Beobachtungszeitraum von 1998 bis 2021 wurden insgesamt rund 7,3 Mio. Sterbefälle mit einem Sterbealter zwischen 0 und 74 Jahren verzeichnet. Im Durchschnitt gab es etwa 305.000 vorzeitige Sterbefälle pro Jahr, wobei die Werte bei den Männern höher lagen als bei den Frauen (Tab. 1). Abb. 1 zeigt die altersstandardisierte Mortalitätsrate für die Bevölkerung im Alter unter 75 Jahren differenziert nach dem Grad regionaler sozioökonomischer Deprivation im Jahr 2019. Bei Männern und Frauen zeichnet sich jeweils das Muster eines fein abgestuften sozialen Gradienten ab: Je höher die sozioökonomische Deprivation der Wohnregion, desto höher lag die vorzeitige Sterblichkeit. Männer im höchsten Deprivationsquintil hatten ein 43 % höheres Risiko, vor einem Alter von 75 Jahren zu versterben, als Gleichaltrige im niedrigsten Deprivationsquintil (SMR = 1,43). Bei Frauen war dieses Risiko um 33 % erhöht (SMR = 1,33).

**Table 1:** 

	Männer			Frauen		
Deprivation	Kreise	Bevölkerung	Sterbefälle	Kreise	Bevölkerung	Sterbefälle
Quintil 1 – niedrig	80	9.181.009	39.729	80	9.133.438	23.257
Quintil 2	80	7.548.073	36.709	80	7.489.832	20.893
Quintil 3	80	7.438.728	38.316	80	7.331.650	21.492
Quintil 4	80	6.850.082	38.891	80	6.754.164	21.618
Quintil 5 – hoch	80	6.550.758	41.463	80	6.423.991	22.494
Gesamt	400	37.568.651	195.107	400	37.133.074	109.754

In Tab. 2 sind die deprivationsspezifischen Kennzahlen der vorzeitigen Sterblichkeit nach Todesursachen dargestellt. Die vorzeitige Sterblichkeit aufgrund von Krebserkrankungen überstieg im Jahr 2019 sowohl bei Männern als auch bei Frauen in allen Deprivationsquintilen die vorzeitige Sterblichkeit aufgrund von Herz-Kreislauf-Erkrankungen. Der soziale Gradient zuungunsten von Personen in sozioökonomisch höher deprivierten Regionen zeigt sich in der Herz-Kreislauf- und Krebsmortalität, ist aber auch für andere Ursachen vorzeitiger Sterblichkeit deutlich zu erkennen.

**Table 2:** 

	Herz-Kreislauf		Krebs		Andere Ursachen	
	Rate	SMR	Rate	SMR	Rate	SMR
*Männer*						
Quintil 1 – niedrige Deprivation	97	Ref.	142	Ref.	146	Ref.
Quintil 2	109	1,11	157	1,10	160	1,10
Quintil 3	113	1,16	170	1,20	168	1,15
Quintil 4	128	1,31	180	1,27	184	1,26
Quintil 5 – hohe Deprivation	146	1,50	202	1,43	205	1,40
*Frauen*						
Quintil 1 – niedrige Deprivation	37	Ref.	106	Ref.	77	Ref.
Quintil 2	44	1,18	113	1,06	83	1,08
Quintil 3	45	1,21	119	1,12	84	1,09
Quintil 4	52	1,39	122	1,15	88	1,14
Quintil 5 – hohe Deprivation	57	1,53	134	1,27	101	1,32

*Rate* standardisierte Mortalitätsrate (Sterbefälle pro 100.000 Personen), *SMR* Standardized Mortality Ratio (relatives Risiko), *Ref.* Referenzkategorie

**Figure 2:**
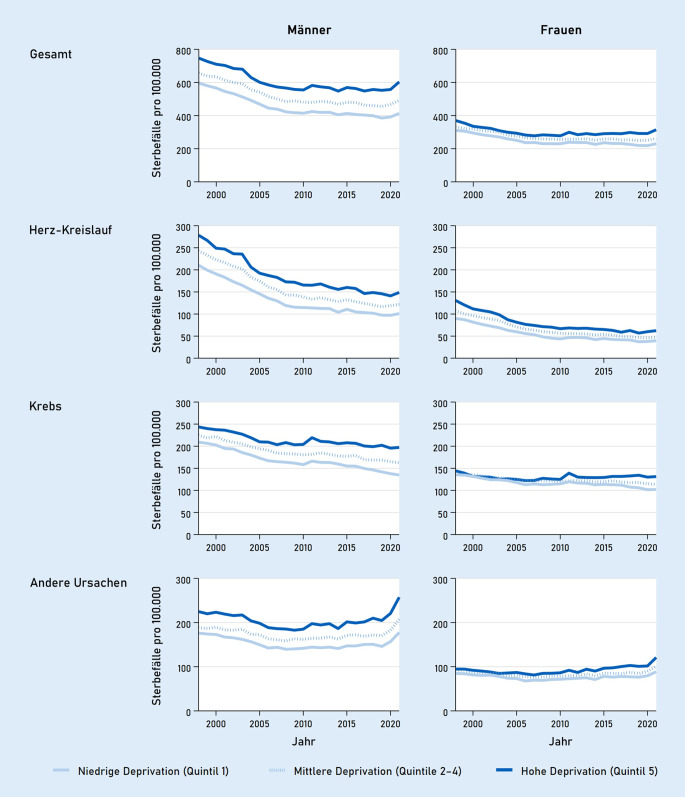

Höhere Mortalitätsraten in höher deprivierten Regionen zeigten sich über den gesamten Beobachtungszeitraum (Abb. 2). Während die vorzeitige Sterblichkeit bis etwa Mitte der 2000er-Jahre in allen Deprivationsquintilen deutlich zurückging, schwächte sich dieser Trend in den Folgejahren deutlich ab. Die Ergebnisse der Joinpoint-Regressionen (siehe Online-Zusatzmaterial 1) zeigen in den 2010er-Jahren nur noch in weniger deprivierten Regionen statistisch signifikante Mortalitätsrückgänge. Dagegen zeichnete sich bei Männern in hoch deprivierten Regionen bis zum Beginn der COVID-19-Pandemie kein signifikanter Rückgang mehr ab und die Raten bei Frauen stiegen in hoch deprivierten Regionen wieder an. Dadurch kam es insgesamt zu einer Ausweitung der regionalen sozioökonomischen Ungleichheit in der vorzeitigen Sterblichkeit über die Zeit, die sich sowohl in gestiegenen Ratendifferenzen (absolute Ungleichheit) als auch gestiegenen Ratenverhältnissen (relative Ungleichheit) zwischen höchstem und niedrigstem Deprivationsquintil widerspiegelt (siehe Online-Zusatzmaterial 2). In den ersten Jahren der COVID-19-Pandemie stieg die vorzeitige Sterblichkeit insgesamt an. Dabei fielen die Anstiege in hoch deprivierten Regionen stärker aus als in niedrig deprivierten Regionen.

Wird zwischen den beiden häufigsten Gruppen von Todesursachen differenziert, zeigen sich unterschiedliche Trends. So sind für die Herz-Kreislauf-Mortalität in allen Deprivationsquintilen deutliche Rückgänge im Beobachtungszeitraum zu verzeichnen, die in hoch deprivierten Regionen absolut gesehen etwas stärker ausfielen als in niedrig deprivierten Regionen. So kam es bei der Herz-Kreislauf-Mortalität zu einer Verringerung der Ratendifferenz (absoluten Ungleichheit) zwischen dem höchsten und niedrigsten Deprivationsquintil. Die Ratenverhältnisse (relative Ungleichheit) weiteten sich angesichts des insgesamt niedrigeren Niveaus der Raten jedoch aus. Für die vorzeitige Krebsmortalität hingegen fielen die Rückgänge in hoch deprivierten Regionen deutlich schwächer aus als in niedrig deprivierten Regionen. Bei Frauen stieg die vorzeitige Krebsmortalität ab etwa Mitte der 2000er-Jahre in hoch deprivierten Regionen sogar leicht an. Dadurch weiteten sich die Ungleichheiten in der Krebsmortalität sowohl in absoluter als auch relativer Hinsicht aus. Für die vorzeitige Sterblichkeit aufgrund anderer Todesursachen waren die Raten ebenfalls bis in die Mitte der 2000er-Jahre in allen Deprivationskategorien rückläufig, während danach Anstiege zu verzeichnen sind, die in hoch deprivierten Regionen durchschnittlich höher ausfielen als in niedrig deprivierten Regionen.

In Tab. 3 sind die Ergebnisse der What-if-Analyse gemäß den 3 oben beschriebenen Szenarien ausgewiesen. Für Szenario 1 zeigen die Ergebnisse das größte Potenzial zur Reduktion der vorzeitigen Sterblichkeit durch eine Verminderung der Mortalitätsungleichheiten: Hätten die Menschen in allen Regionen Deutschlands das gleiche Sterberisiko wie jene in Regionen des niedrigsten Deprivationsquintils, wären im Jahr 2019 insgesamt 35.119 vorzeitige Sterbefälle weniger zu verzeichnen gewesen. Dies entspräche einer Reduktion der vorzeitigen Sterblichkeit um insgesamt 12,8 % bzw. um durchschnittlich etwa einen Sterbefall alle Viertelstunde. Unter Männern fiele die Mortalitätsreduktion etwas höher aus (14,0 %) als unter Frauen (10,6 %). Würde das Sterberisiko in höher deprivierten Regionen auf das Niveau in den sozioökonomisch nächst bessergestellten Regionen gesenkt (Szenario 2), läge die relative Mortalitätsreduktion im Alter unter 75 Jahren bei etwa 6,5 % für Männer und 4,8 % für Frauen. In Szenario 3, das eine Senkung der vorzeitigen Sterblichkeit in Regionen mit überdurchschnittlicher Deprivation auf das Niveau in Regionen mit mittlerer Deprivation vorsieht, betrügen diese Werte 3,9 % und 2,7 %.

**Table 3:** 

	Anzahl der vorzeitigen Sterbefälle			
	Real	Szenario 1	Szenario 2	Szenario 3
	Beobachtet	Erwartet	Erwartet	Erwartet
*Männer*				
Quintil 1 – niedrige Deprivation	36.600	36.600	36.600	36.600
Quintil 2	35.427	32.046	32.046	35.427
Quintil 3	42.389	36.052	39.849	42.389
Quintil 4	40.601	31.735	37.259	37.259
Quintil 5 – hohe Deprivation	18.817	13.040	16.702	15.319
Insgesamt	173.834	149.474	162.456	166.994
Differenz^a^	–	−24.360	−11.378	−6840
Anteil^b^		−14,0 %	−6,5 %	−3,9 %
*Frauen*				
Quintil 1 – niedrige Deprivation	22.314	22.314	22.314	22.314
Quintil 2	21.140	19.375	19.375	21.140
Quintil 3	24.494	21.698	23.674	24.494
Quintil 4	22.691	19.080	21.509	21.509
Quintil 5 – hohe Deprivation	10.488	7901	9397	8908
Insgesamt	101.127	90.368	96.269	98.365
Differenz^a^	–	−10.759	−4858	−2762
Anteil^b^		−10,6 %	−4,8 %	−2,7 %

^a^Differenz zwischen der Summe erwarteter Sterbefälle und der Summe beobachteter Sterbefälle (absolute Mortalitätsreduktion)

^b^Anteil der im Szenario reduzierten Sterbefälle an allen beobachteten Sterbefällen (relative Mortalitätsreduktion)

## Diskussion

Dies ist die erste bundesweite Studie zu regionalen sozioökonomischen Ungleichheiten in der vorzeitigen Sterblichkeit, in der zeitliche Trends, todesursachenspezifische Muster und Potenziale, die mit einer Reduktion der Ungleichheiten verbunden sind, untersucht wurden. Den Ergebnissen zufolge haben Männer und Frauen, die in sozioökonomisch stärker deprivierten Regionen leben, beträchtlich höhere Risiken für vorzeitige Sterblichkeit als jene in wohlhabenderen Regionen. Dieser soziale Gradient zeigt sich nicht nur für die Herz-Kreislauf- und Krebssterblichkeit, sondern auch für andere Todesursachen. Im Zeitverlauf weiteten sich die regionalen sozioökonomischen Mortalitätsungleichheiten aus, was sich schon vor der COVID-19-Pandemie abzeichnete und sich in der Pandemie noch weiter verschärfte. Würden diese Mortalitätsungleichheiten durch Senkung des vorzeitigen Sterberisikos in höher deprivierten Regionen verringert, läge die vorzeitige Sterblichkeit in Deutschland insgesamt – je nach Szenario – um bis zu 13 % niedriger.

Die Ergebnisse stehen im Einklang mit Befunden aus vorherigen Analysen für Deutschland, in denen sozioökonomische Regionalindikatoren, wie z. B. Arbeitslosenquoten oder Pro-Kopf-Einkommen, mit regionalen Mortalitäts- oder Lebenserwartungsdaten in Zusammenhang gesetzt wurden [21, 28, 29]. Ausweitungen sozioökonomischer Mortalitätsungleichheiten wurden hierzulande bereits in Sozialversicherungsdaten gefunden und deuteten sich in der Tendenz auch in Daten aus Bevölkerungsstichproben an [12, 17, 18, 43], sie konnten bislang aber nicht todesursachenspezifisch für Deutschland untersucht werden. International, besonders im Vereinigten Königreich, haben ökologische Studien zum Zusammenhang zwischen regionaler sozioökonomischer Deprivation und Mortalität bereits eine längere Tradition und wiesen schon in den 1980er- und 1990er-Jahren auf Ausweitungen von Ungleichheiten in der vorzeitigen Sterblichkeit hin [44–46]. Das Ergebnis, dass sich die Ausweitung der Mortalitätsungleichheiten in der COVID-19-Pandemie in Deutschland verschärfte, steht im Einklang mit Befunden, die eine höhere COVID-19-assoziierte Mortalität in höher deprivierten Regionen zeigten [22, 47]. Der vorliegende Befund für die vorzeitige Herz-Kreislauf-Sterblichkeit, dass sich absolute Ungleichheiten aufgrund stärkerer Mortalitätsrückgänge in sozial benachteiligten Gruppen verringerten, während relative Ungleichheiten im Zuge dieser Entwicklung zunahmen, zeigte sich auch in einer großen europäischen Studie auf Basis von Individualdaten [23].

### Stärken und Limitationen

Zu den Vorteilen des ökologischen Studiendesigns für die Untersuchung sozioökonomischer Mortalitätsungleichheiten zählt, dass durch dieses Vorgehen die amtliche Todesursachenstatistik und damit eine Vollerhebung der Sterbefälle in Deutschland für sozialepidemiologische Analysen nutzbar gemacht werden. Die Todesursachenstatistik ermöglicht es, entsprechende Analysen nicht nur für die Gesamtmortalität, sondern auch für verschiedene Todesursachen bundesweit durchzuführen. Dies sind wichtige Vorteile gegenüber den eingangs erwähnten Alternativmöglichkeiten für Ungleichheitsanalysen zur Mortalität in Deutschland wie jenen auf Basis von Bevölkerungsstichproben, z. B. des Sozio-oekonomischen Panels (SOEP), oder Routinedaten der Sozialversicherungen. Denn weder die SOEP-Daten noch die Sozialversicherungsdaten enthalten direkte Informationen zur Todesursache. Zudem bleiben die Analysen der Sozialversicherungsdaten auf die Personenkreise gesetzlich rentenversicherter Männer oder Versicherter bestimmter gesetzlicher Krankenkassen beschränkt [16–18], während die Todesursachenstatistik Aussagen über die Gesamtbevölkerung erlaubt. Die Vorteile der Bevölkerungsstichproben- und Sozialversicherungsdaten liegen hingegen darin, dass sie sozioökonomische Individualdaten enthalten und die sozioökonomische Position deswegen nicht über sozialräumliche Daten approximiert werden muss, wie es im ökologischen Design erfolgt.

Die sozialräumliche Approximation der sozioökonomischen Position, die in der vorliegenden Studie anhand des GISD auf Kreisebene erfolgte (ökologisches Studiendesign), ist insbesondere mit der Limitation verbunden, dass ein ökologischer Fehlschluss nicht ausgeschlossen werden kann. Demnach beschränkt sich der Erkenntniswert der Studie auf die Beschreibung raumbezogener Mortalitätsungleichheiten und Identifikation benachteiligter Regionen; Rückschlüsse auf kausale Wirkungen können nicht gezogen werden [19]. Dabei ist auch zu berücksichtigen, dass Heterogenität innerhalb von bevölkerungsreichen Land- bzw. Stadtkreisen, z. B. zwischen Bezirken innerhalb Berlins, anhand der verwendeten Daten nicht abgebildet werden konnte. Eine weitere Limitation besteht darin, dass die Todesursachenstatistik nur das zum Tode führende Grundleiden als Information zur Todesursache enthält. Insbesondere bei Periodenereignissen wie der COVID-19-Pandemie könnte es zu einer Unterschätzung der Mortalität von chronischen Erkrankungen aufgrund selektiver Mortalität kommen. Eine multikausale Todesursachenstatistik, die alle auf der Todesbescheinigung verfügbaren Informationen berücksichtigt, könnte dabei helfen, das Ausmaß der Dynamik in der Mortalität besser abzuschätzen. Die Etablierung einer solchen Statistik steht für Deutschland bundesweit bislang noch aus. Hinsichtlich der What-if-Analyse ist zu berücksichtigen, dass sie auf kontrafaktischen Szenarien basiert und dazu dient, Potenziale von Verringerungen der gesundheitlichen Ungleichheit für die Bevölkerungsgesundheit insgesamt abzuschätzen. Wie dies in der Realität erreicht werden könnte, ist nicht Bestandteil dieses Ansatzes [32]. In Berechnungen für England fiel der Anteil ungleichheitsattributabler vorzeitiger Sterblichkeit insgesamt höher aus als in unseren Berechnungen für Szenario 1. Die Ergebnisse sind jedoch nicht direkt vergleichbar, u. a. weil in der englischen Studie Deprivationsdezile statt -quintile verwendet wurden und dadurch eine größere regionale sozioökonomische Spreizung als in unserer Studie berücksichtigt wurde [25].

### Schlussfolgerungen

Die Verknüpfung der Todesursachenstatistik mit dem GISD auf kleinräumiger Ebene bietet für Deutschland neue Möglichkeiten zur Untersuchung von Art und Ausmaß sozioökonomischer Mortalitätsungleichheiten. Diese bestehen insbesondere darin, dass Langzeittrends der sozioökonomischen Mortalitätsungleichheit seit Ende der 1990er-Jahre todesursachenspezifisch analysiert und Aussagen über die bundesweite Gesamtbevölkerung getroffen werden können. Unter Beachtung der Limitationen des ökologischen Designs kann dieser Ansatz somit einen wichtigen Beitrag dazu leisten, die in Deutschland bestehende Datenlücke zu sozioökonomischen Ungleichheiten in der Mortalität zu überbrücken. Aktuelle Bemühungen, die bestehende Datenlücke zu schließen, z. B. im Rahmen des im Aufbau befindlichen Registerzensus oder der Etablierung eines nationalen Mortalitätsregisters, sollten jedoch nicht aus den Augen verloren werden, um entsprechende Analysemöglichkeiten künftig auch mit Individualdaten schaffen zu können.

Insgesamt bekräftigen die Befunde der vorliegenden Studie, dass die Verbesserung der Gesundheits- und Lebenschancen von Menschen in sozioökonomisch benachteiligten Verhältnissen eine wichtige und politikressortübergreifende Aufgabe bleibt, die mit beträchtlichen Potenzialen für die Gesundheit der Bevölkerung insgesamt und die weitere Reduktion der vorzeitigen Sterblichkeit verbunden ist. Die Herstellung gleichwertiger Lebensverhältnisse im Bundesgebiet, die als politisches Ziel im Grundgesetz verankert ist, stellt angesichts der zunehmenden sozioökonomischen Mortalitätsungleichheit auf regionaler Ebene auch ein wichtiges Handlungsfeld zur Verbesserung der gesundheitlichen Chancengleichheit in Deutschland dar.

### Supplementary Information






## References

[1] Mackenbach JP, Stirbu I, Roskam A-J (2008). Socioeconomic inequalities in health in 22 European countries. N Engl J Med.

[2] Marmot M, Allen J, Goldblatt P (2010). Fair society, healthy lives. The Marmot Review. Strategic review of health inequalities in England post-2010.

[3] Commission on Social Determinants of Health (2008). Closing the gap in a generation: health equity through action on the social determinants of health. Final Report of the Commission on Social Determinants of Health.

[4] Lampert T, Richter M, Schneider S, Spallek J, Dragano N (2016). Soziale Ungleichheit und Gesundheit: Stand und Perspektiven der sozialepidemiologischen Forschung in Deutschland. Bundesgesundheitsbl.

[5] Lampert T, Hoebel J, Kuntz B, Müters S, Kroll LE (2017). Gesundheitliche Ungleichheit in verschiedenen Lebensphasen.

[6] Mielck A, Wild V (2021). Gesundheitliche Ungleichheit – Auf dem Weg von Daten zu Taten: Fragen und Empfehlungen aus Sozial-Epidemiologie und Public-Health-Ethik.

[7] Kröger H, Pakpahan E, Hoffmann R (2015). What causes health inequality? A systematic review on the relative importance of social causation and health selection. Eur J Public Health.

[8] Chetty R, Stepner M, Abraham S (2016). The association between income and life expectancy in the United States, 2001–2014. JAMA.

[9] Murtin F, Mackenbach J, Jasilionis D, d’Ercole MM (2017). Inequalities in longevity by education in OECD countries. OECD Statistics Working Papers, No. 2017/02.

[10] Mackenbach JP (2019). Health Inequalities: Persistence and change in European Welfare States.

[11] Lampert T, Hoebel J, Kroll LE, Luy M (2018). Soziale Unterschiede in der Lebenserwartung. Public Health Forum.

[12] Lampert T, Hoebel J, Kroll LE (2019). Social differences in mortality and life expectancy in Germany: current situation and trends. J Health Monit.

[13] Luy M, Wegner-Siegmundt C, Wiedemann A, Spijker J (2015). Life Expectancy by Education, Income and Occupation in Germany: Estimations Using the Longitudinal Survival Method. Comp Popul Stud.

[14] Lampert T, Kroll LE (2014). Soziale Unterschiede in der Mortalität und Lebenserwartung. GBE kompakt.

[15] Hoebel J, Müters S (im Druck) Sozioökonomischer Status und Gesundheit – Datenlage, Befunde und Entwicklungen in Deutschland. WSI-Mitteilungen

[16] Grigoriev P, Scholz R, Shkolnikov VM (2019). Socioeconomic differences in mortality among 27 million economically active Germans: a cross-sectional analysis of the German Pension Fund data. BMJ Open.

[17] Tetzlaff F, Epping J, Sperlich S, Tetzlaff J (2020). Widening income inequalities in life expectancy? Analysing time trends based on German health insurance data. J Epidemiol Community Health.

[18] Wenau G, Grigoriev P, Shkolnikov V (2019). Socioeconomic disparities in life expectancy gains among retired German men, 1997–2016. J Epidemiol Community Health.

[19] Michalski N, Reis M, Tetzlaff F (2022). German Index of Socioeconomic Deprivation (GISD): Revision, Aktualisierung und Anwendungsbeispiele. J Health Monit.

[20] Tetzlaff F, Nowossadeck E, Jansen L (2023). Widening area-based socioeconomic inequalities in cancer mortality in Germany between 2003 and 2019. Sci Rep.

[21] Kibele EUB (2012). Regional Mortality Differences in Germany.

[22] Hoebel J, Michalski N, Diercke M (2021). Emerging socio-economic disparities in COVID-19-related deaths during the second pandemic wave in Germany. Int J Infect Dis.

[23] Mackenbach JP, Kulhánová I, Menvielle G (2015). Trends in inequalities in premature mortality: a study of 3.2 million deaths in 13 European countries. J Epidemiol Community Health.

[24] Németh N, Boncz I, Pakai A (2023). Inequalities in premature mortality from ischaemic heart disease in the WHO European region. Cent Eur J Public Health.

[25] Lewer D, Jayatunga W, Aldridge RW (2020). Premature mortality attributable to socioeconomic inequality in England between 2003 and 2018: an observational study. Lancet Public Health.

[26] Kinge JM, Modalsli JH, Øverland S (2019). Association of Household Income With Life Expectancy and Cause-Specific Mortality in Norway, 2005–2015. JAMA.

[27] Kroll LE, Schumann M, Hoebel J (2017). Regionale Unterschiede in der Gesundheit: Entwicklung eines sozioökonomischen Deprivationsindex für Deutschland. J Health Monit.

[28] Plümper T, Laroze D, Neumayer E (2018). The limits to equivalent living conditions: regional disparities in premature mortality in Germany. Z Gesundh Wiss.

[29] Rau R, Schmertmann CP (2020). District-level life expectancy in Germany. Dtsch Ärztebl Int.

[30] Michelsen K, Brand H (2012). „Gesundheit 2020“ – das neue europäische Rahmenkonzept der WHO. Gesundheitswesen.

[31] Zukunftsforum Public Health (2021). Eckpunkte einer Public-Health-Strategie für Deutschland.

[32] Mackenbach JP, Meerding WJ, Kunst AE (2011). Economic costs of health inequalities in the European Union. J Epidemiol Community Health.

[33] Forschungsdatenzentren der Statistischen Ämter des Bundes und der Länder (2021). Todesursachenstatistik.

[34] Organisation for Economic Co-operation and Development, European Commission (2022) Avoidable mortality: OECD/Eurostat lists of preventable and treatable causes of death (January 2022 version). https://www.oecd.org/health/health-systems/Avoidable-mortality-2019-Joint-OECD-Eurostat-List-preventable-treatable-causes-of-death.pdf. Zugegriffen: 6. Okt. 2023

[35] Stolpe S, Kowall B, Stang A (2023). The Quality of Cause-Of-Death Statistics After the Introduction of the Electronic Coding System Iris/Muse-an Analysis of Mortality Data, 2005–2019. Dtsch Ärztebl Int.

[36] Wengler A, Rommel A, Plaß D (2019). ICD-Codierung von Todesursachen: Herausforderungen bei der Berechnung der Krankheitslast in Deutschland. Bundesgesundheitsbl.

[37] Michalski N, Reis M, Tetzlaff F, Nowossadeck E, Hoebel J (2022). German Index of Socioeconomic Deprivation (GISD).

[38] Eurostat (2013). Revision of the European Standard Population: Report of Eurostat’s task force.

[39] Kim HJ, Chen HS, Byrne J, Wheeler B, Feuer EJ (2022). Twenty years since Joinpoint 1.0: Two major enhancements, their justification, and impact. Stat Med.

[40] Zhang NR, Siegmund DO (2007). A modified Bayes information criterion with applications to the analysis of comparative genomic hybridization data. Biometrics.

[41] Whitehead M, Dahlgren G (2006). Levelling up (part 1): a discussion paper on concepts and principles for tackling social inequities in health.

[42] Ezzati M, Lopez AD, Rodgers A, Vander Hoorn S, Murray CJ (2002). Selected major risk factors and global and regional burden of disease. Lancet.

[43] Kibele EUB, Jasilionis D, Shkolnikov VM (2013). Widening socioeconomic differences in mortality among men aged 65 years and older in Germany. J Epidemiol Community Health.

[44] Eames M, Ben-Shlomo Y, Marmot M (1993). Social Deprivation and Premature Mortality: Regional Comparison Across England. BMJ.

[45] Higgs G, Senior ML, Williams HC (1998). Spatial and temporal variation of mortality and deprivation. 1: widening health inequalities. Environ Plan A.

[46] Sloggett A, Joshi H (1994). Higher mortality in deprived areas: community or personal disadvantage?. BMJ.

[47] Hoebel J, Haller S, Bartig S (2022). Soziale Ungleichheit und COVID-19 in Deutschland – Wo stehen wir in der vierten Pandemiewelle?. Epidemiol Bull.

