# Supplementary material for: Socioeconomic deprivation and premature mortality in Germany, 1998–2021: An ecological study with what-if scenarios of inequality reduction
Source: Bundesgesundheitsblatt Gesundheitsforschung Gesundheitsschutz. 2024 Apr 8;67(5):528–37. [Article in German] doi: 10.1007/s00103-024-03862-0 (PMC11093858; doi:10.1007/s00103-024-03862-0)
Supplement: Supplementary file 1 [file 103_2024_3862_MOESM1_ESM.pdf]

Online-Zusatzmaterial 1

Auf den folgenden Seiten sind die Ergebnisse der Joinpoint-Regressionen für die zeitlichen Trends der altersstandardisierten Mortalitätsraten vor einem Alter von 75 Jahren stratifiziert nach Geschlecht und regionaler sozioökonomischer Deprivation sowie Todesursachen dargestellt. In den einzelnen Abbildungen sind oben links die Werte des zeitlichen Trends in der identifizierten Periode als Annual Percent Change (APC) ausgewiesen.

Inhaltsübersicht:

- Seite 2: Vorzeitige Sterblichkeit (Todesursachen: alle) – Männer
- Seite 3: Vorzeitige Sterblichkeit (Todesursachen: alle) – Frauen

- Seite 4: Vorzeitige Sterblichkeit (Todesursachen: Herz-Kreislauf-Erkrankungen) – Männer
- Seite 5: Vorzeitige Sterblichkeit (Todesursachen: Herz-Kreislauf-Erkrankungen) – Frauen

- Seite 6: Vorzeitige Sterblichkeit (Todesursachen: Krebserkrankungen) – Männer
- Seite 7: Vorzeitige Sterblichkeit (Todesursachen: Krebserkrankungen) – Frauen
-
- Seite 8: Vorzeitige Sterblichkeit (Todesursachen: andere) – Männer
- Seite 9: Vorzeitige Sterblichkeit (Todesursachen: andere) – Frauen

Todesursachen: alle

Männer – niedrige Deprivation:

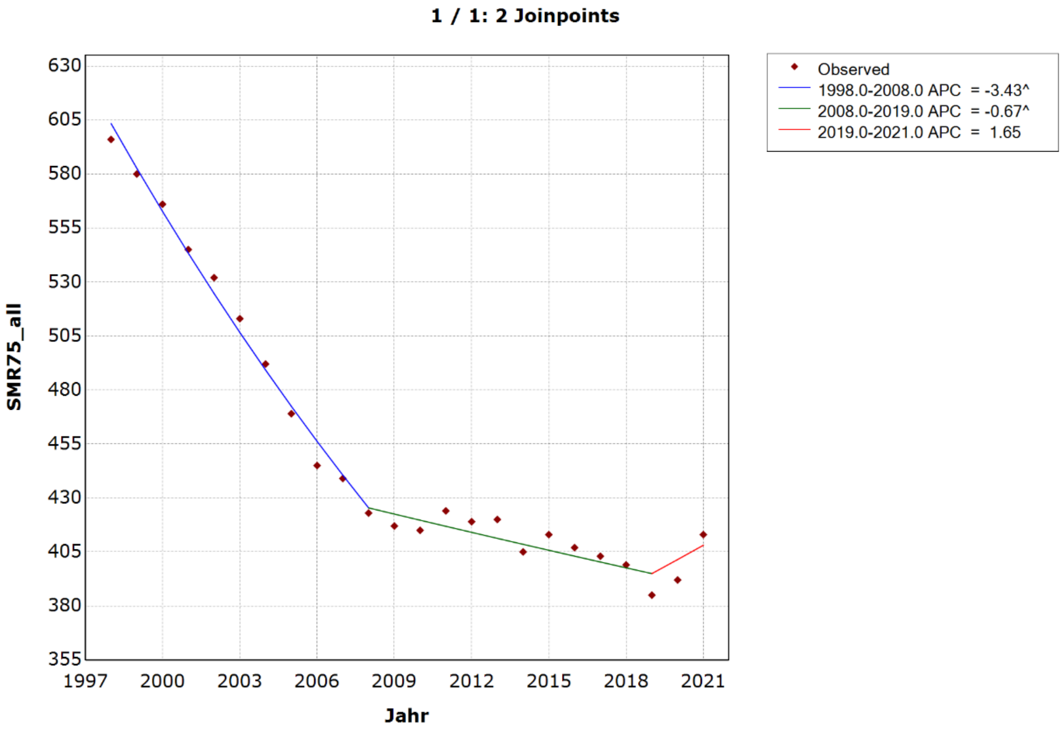

^ Indicates that the Annual Percent Change (APC) is significantly different from zero at the alpha = 0.05 level.
Final Selected Model: 2 Joinpoints.

Männer – hohe Deprivation:

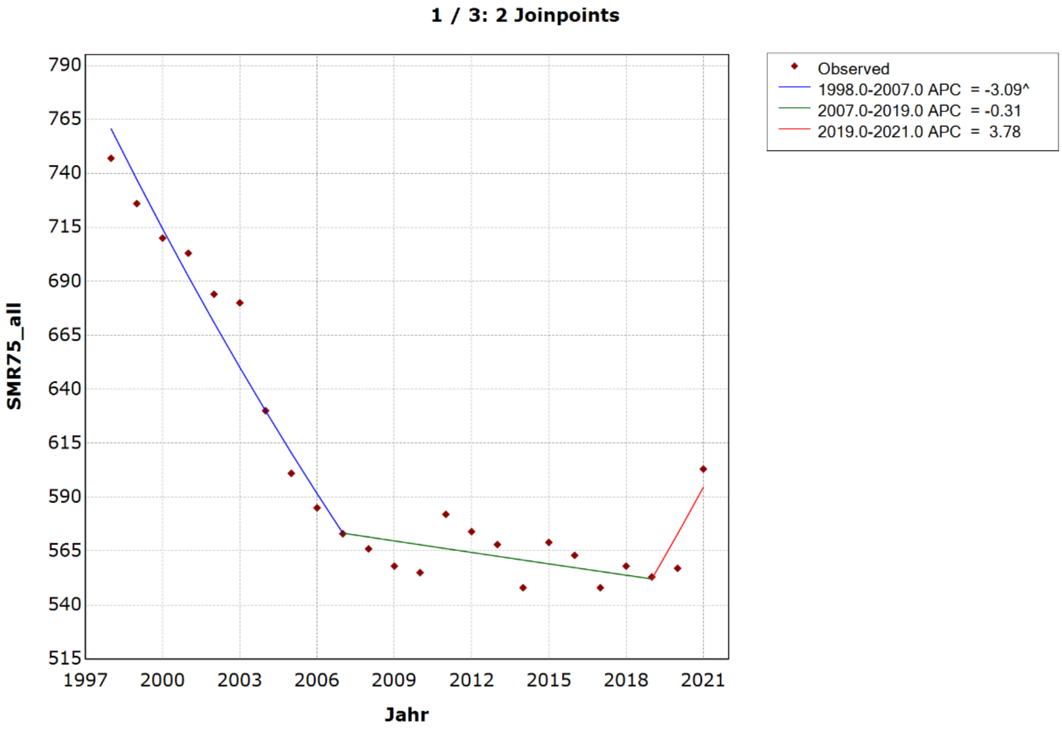

^ Indicates that the Annual Percent Change (APC) is significantly different from zero at the alpha = 0.05 level.
Final Selected Model: 2 Joinpoints.

Todesursachen: alle

Frauen – niedrige Deprivation:

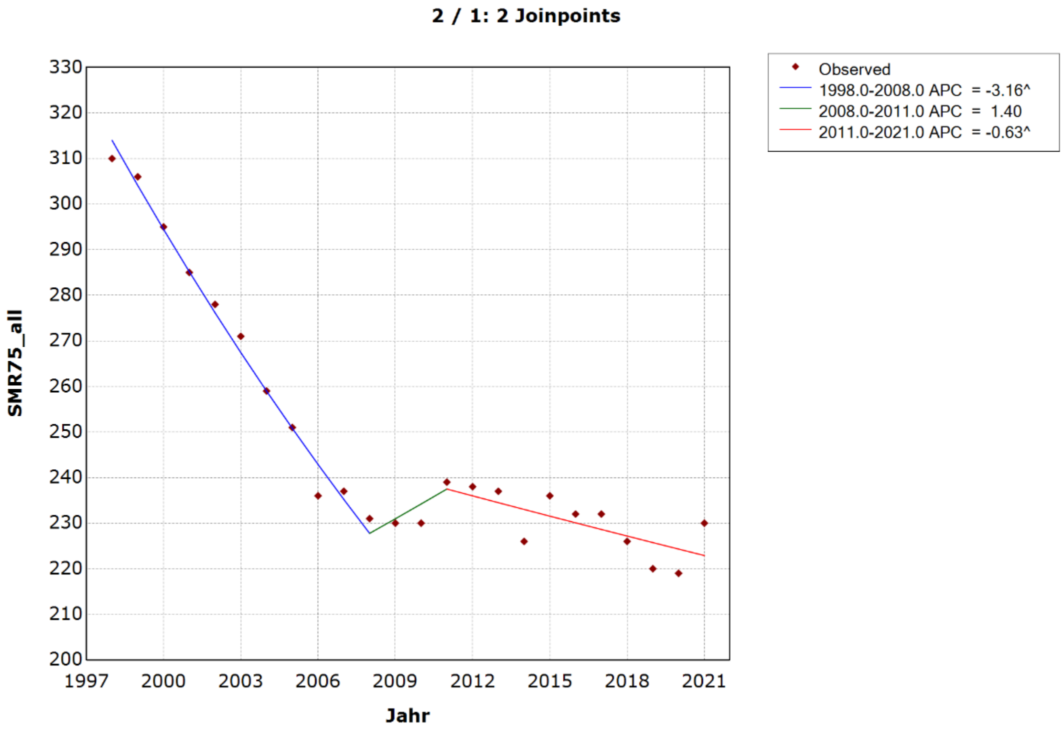

^ Indicates that the Annual Percent Change (APC) is significantly different from zero at the alpha = 0.05 level.
Final Selected Model: 2 Joinpoints.

Frauen – hohe Deprivation:

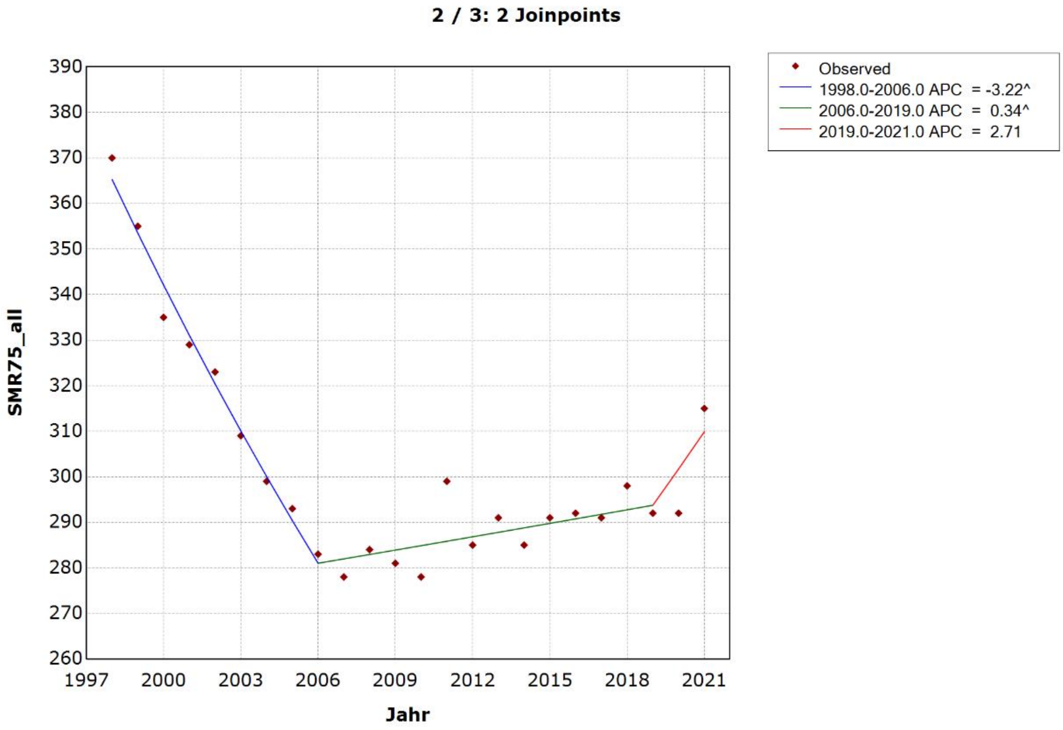

^ Indicates that the Annual Percent Change (APC) is significantly different from zero at the alpha = 0.05 level.
Final Selected Model: 2 Joinpoints.

Todesursachen: Herz-Kreislauf-Erkrankungen

Männer – niedrige Deprivation:

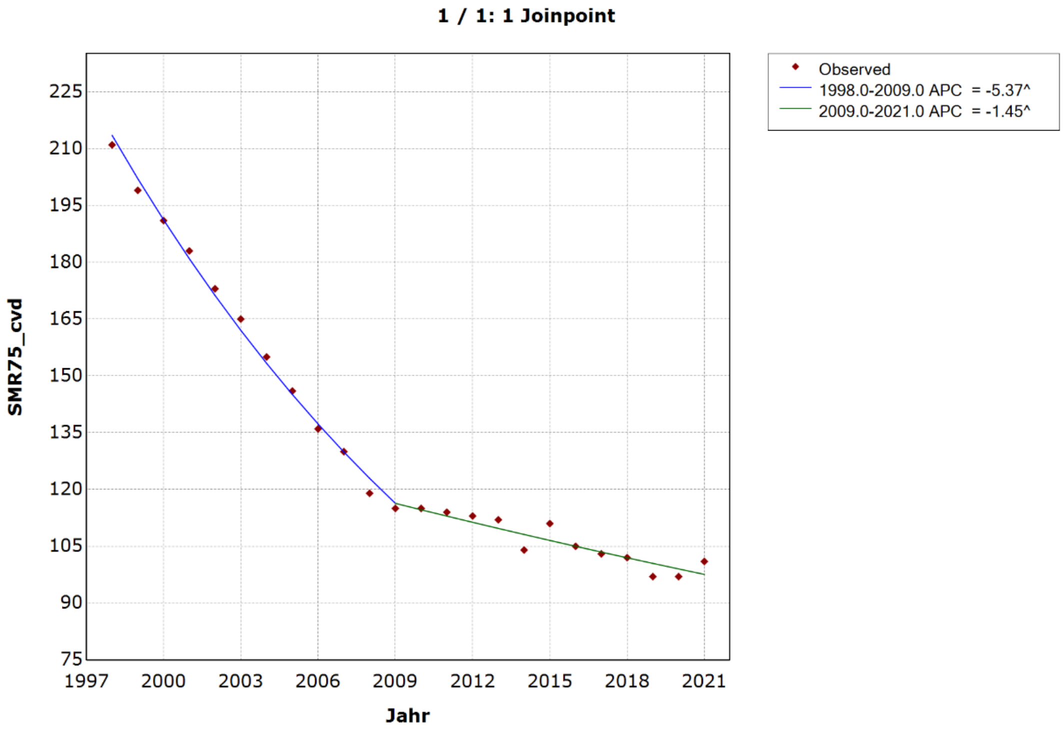

^ Indicates that the Annual Percent Change (APC) is significantly different from zero at the alpha = 0.05 level.
Final Selected Model: 1 Joinpoint.

Männer – hohe Deprivation:

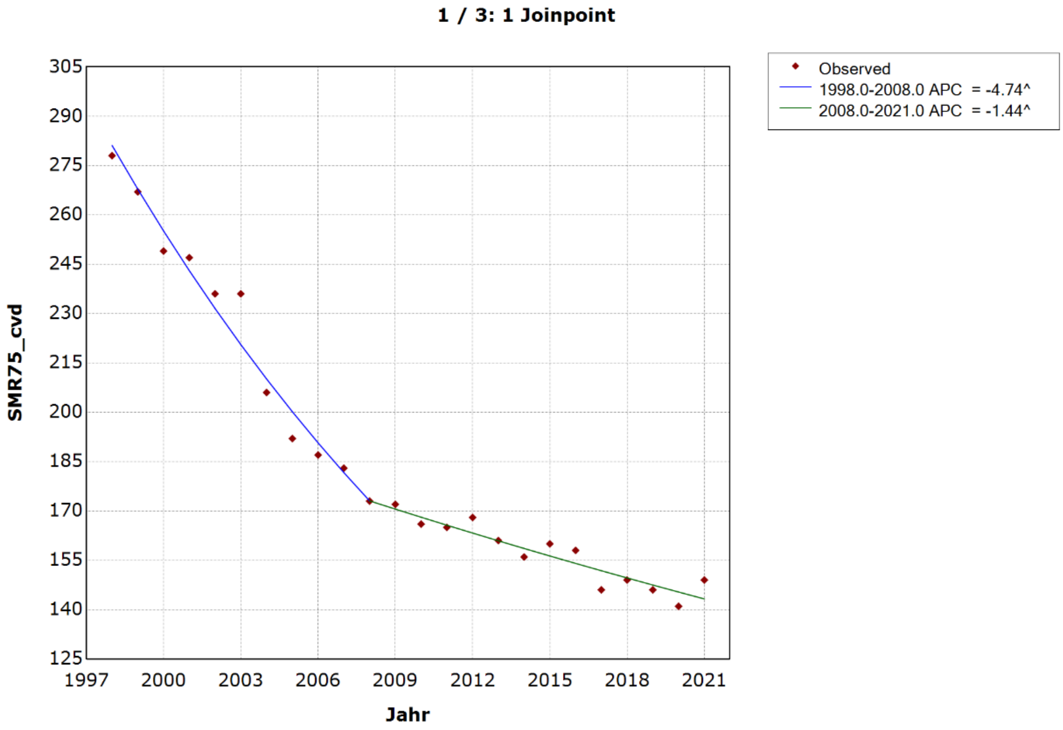

^ Indicates that the Annual Percent Change (APC) is significantly different from zero at the alpha = 0.05 level.
Final Selected Model: 1 Joinpoint.

Todesursachen: Herz-Kreislauf-Erkrankungen

Frauen – niedrige Deprivation:

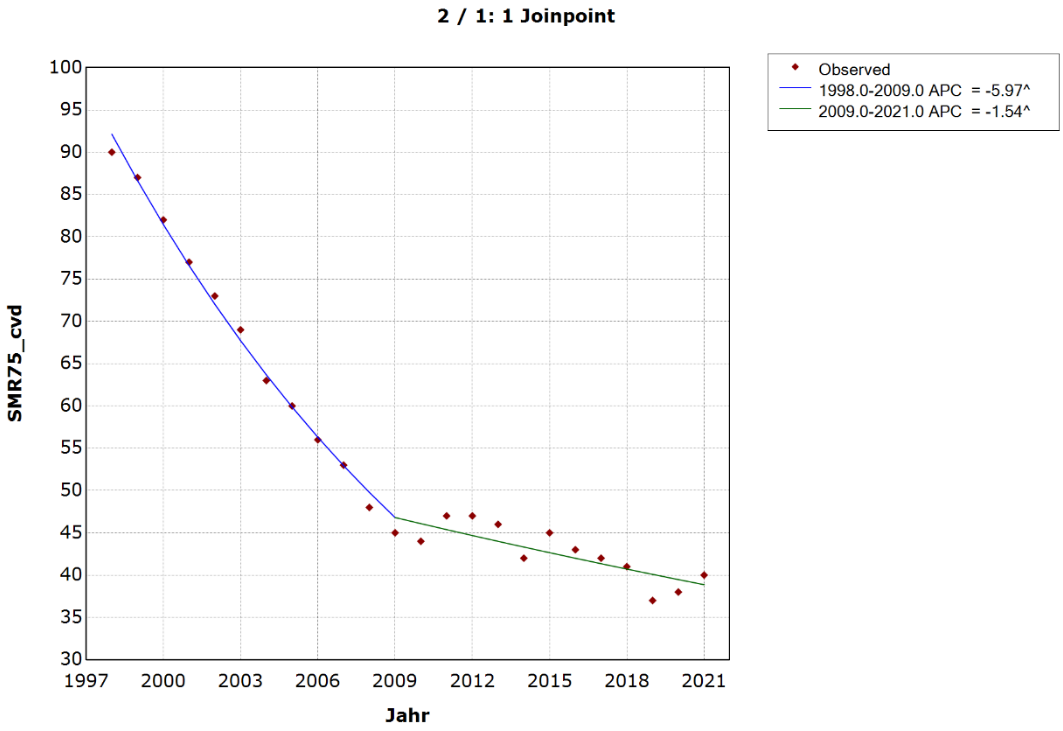

^ Indicates that the Annual Percent Change (APC) is significantly different from zero at the alpha = 0.05 level.
Final Selected Model: 1 Joinpoint.

Frauen – hohe Deprivation:

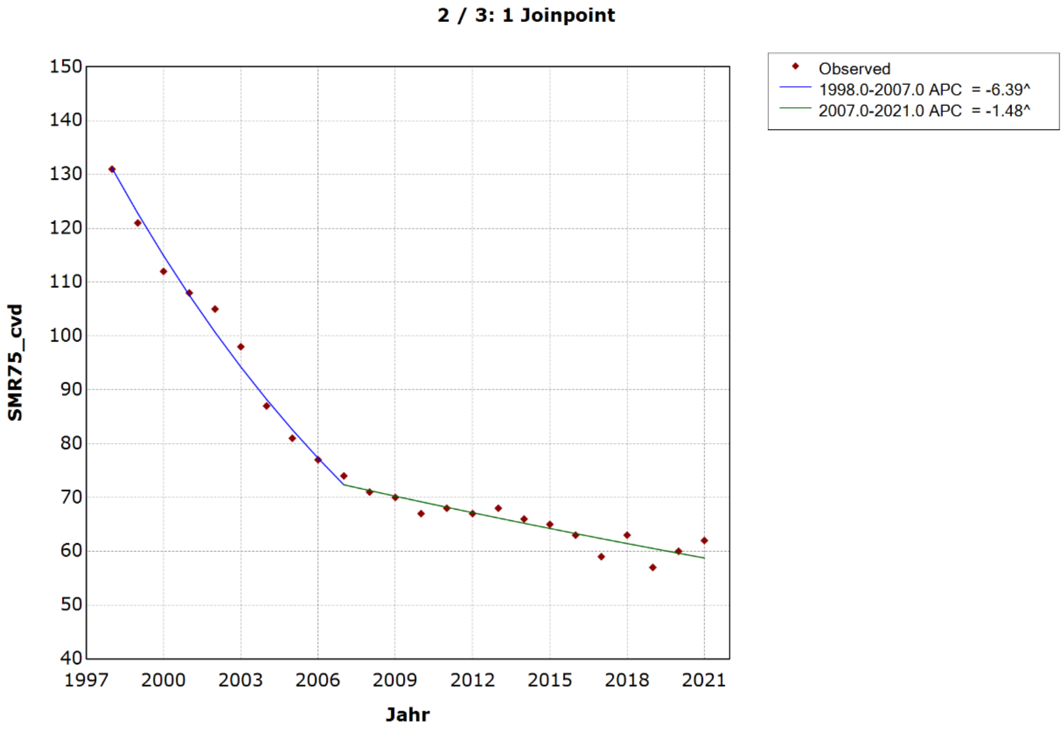

^ Indicates that the Annual Percent Change (APC) is significantly different from zero at the alpha = 0.05 level.
Final Selected Model: 1 Joinpoint.

Todesursachen: Krebserkrankungen

Männer – niedrige Deprivation:

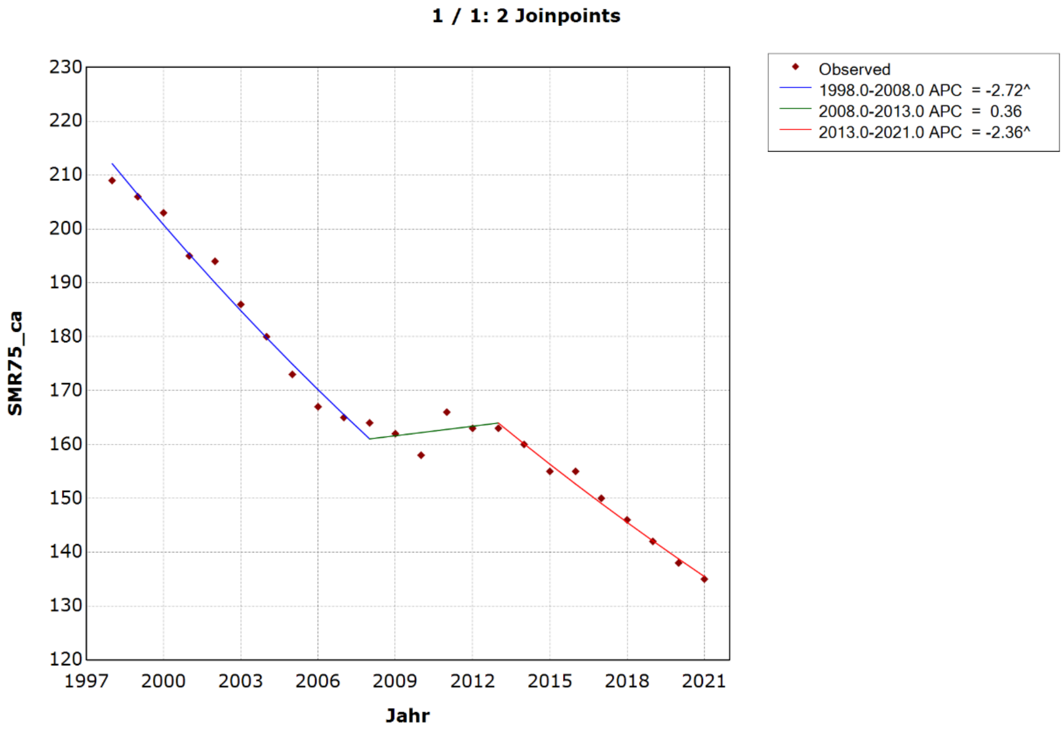

^ Indicates that the Annual Percent Change (APC) is significantly different from zero at the alpha = 0.05 level.
Final Selected Model: 2 Joinpoints.

Männer – hohe Deprivation:

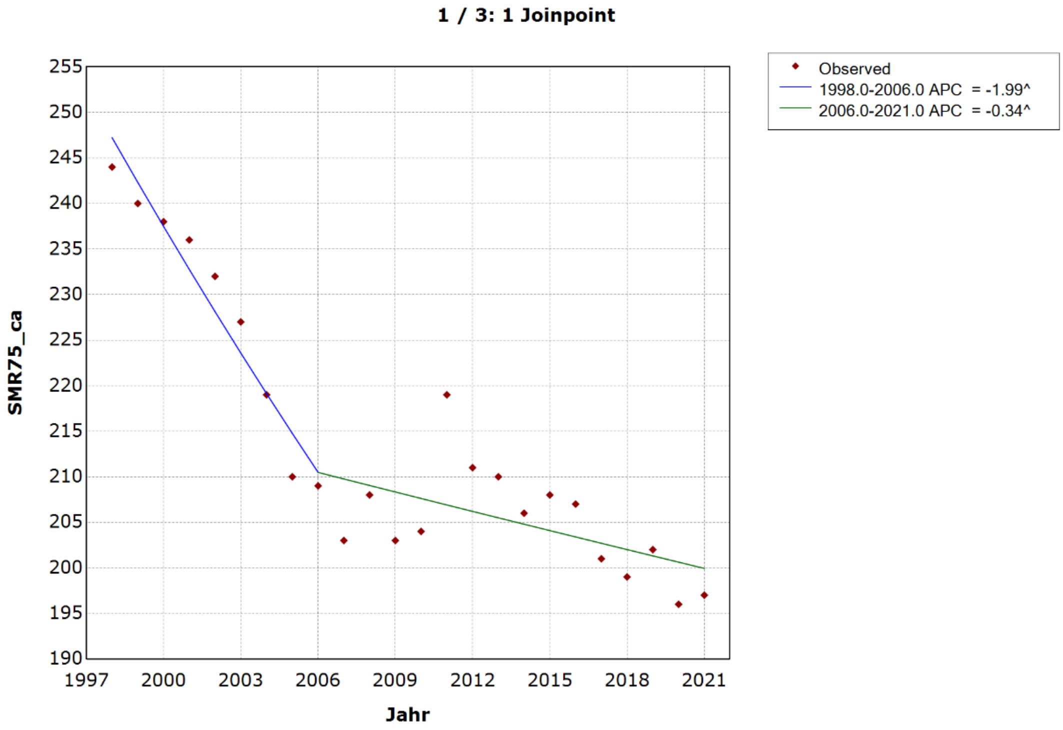

^ Indicates that the Annual Percent Change (APC) is significantly different from zero at the alpha = 0.05 level.
Final Selected Model: 1 Joinpoint.

Todesursachen: Krebserkrankungen

Frauen – niedrige Deprivation:

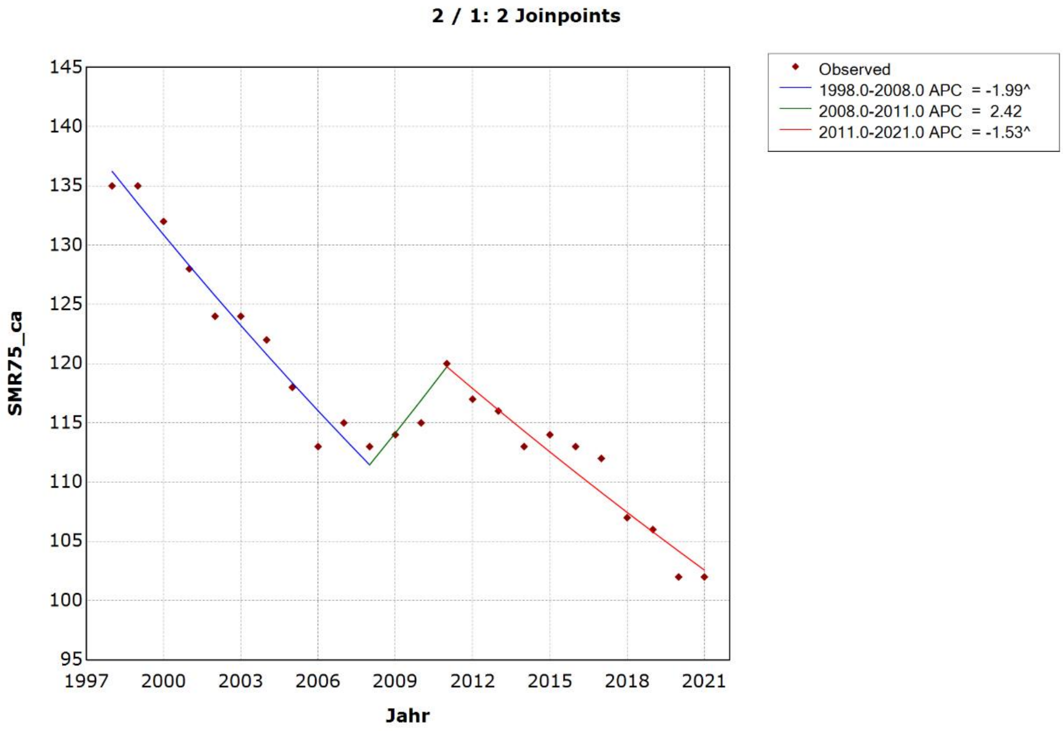

^ Indicates that the Annual Percent Change (APC) is significantly different from zero at the alpha = 0.05 level.
Final Selected Model: 2 Joinpoints.

Frauen – hohe Deprivation:

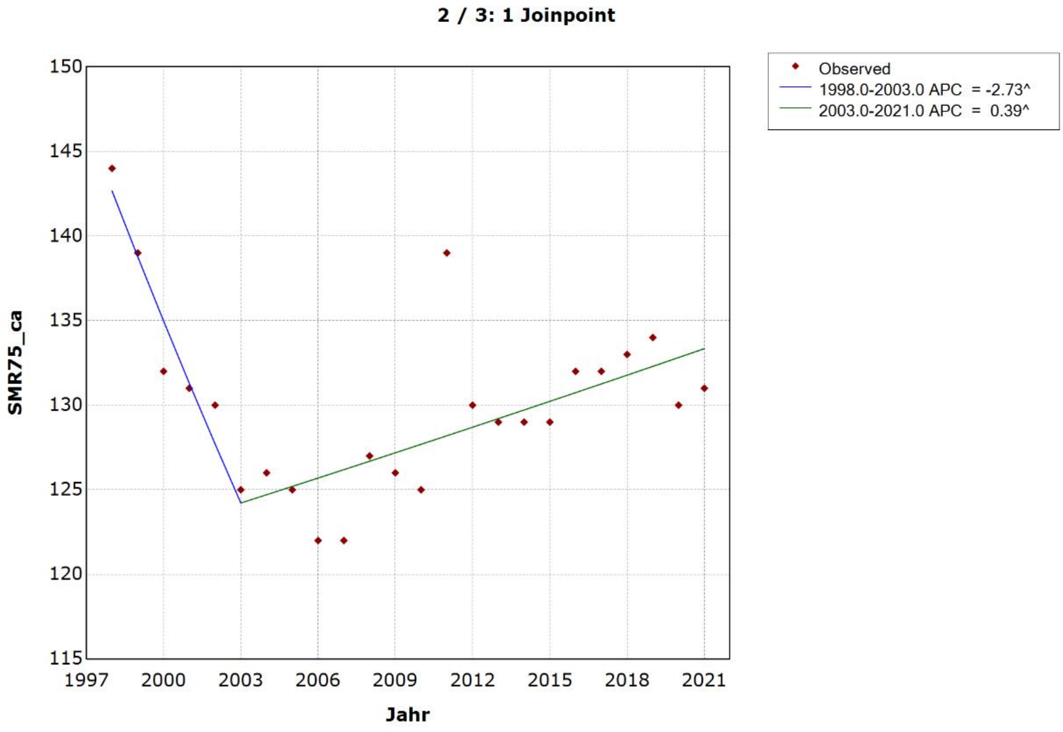

^ Indicates that the Annual Percent Change (APC) is significantly different from zero at the alpha = 0.05 level.
Final Selected Model: 1 Joinpoint.

Todesursachen: andere

Männer – niedrige Deprivation:

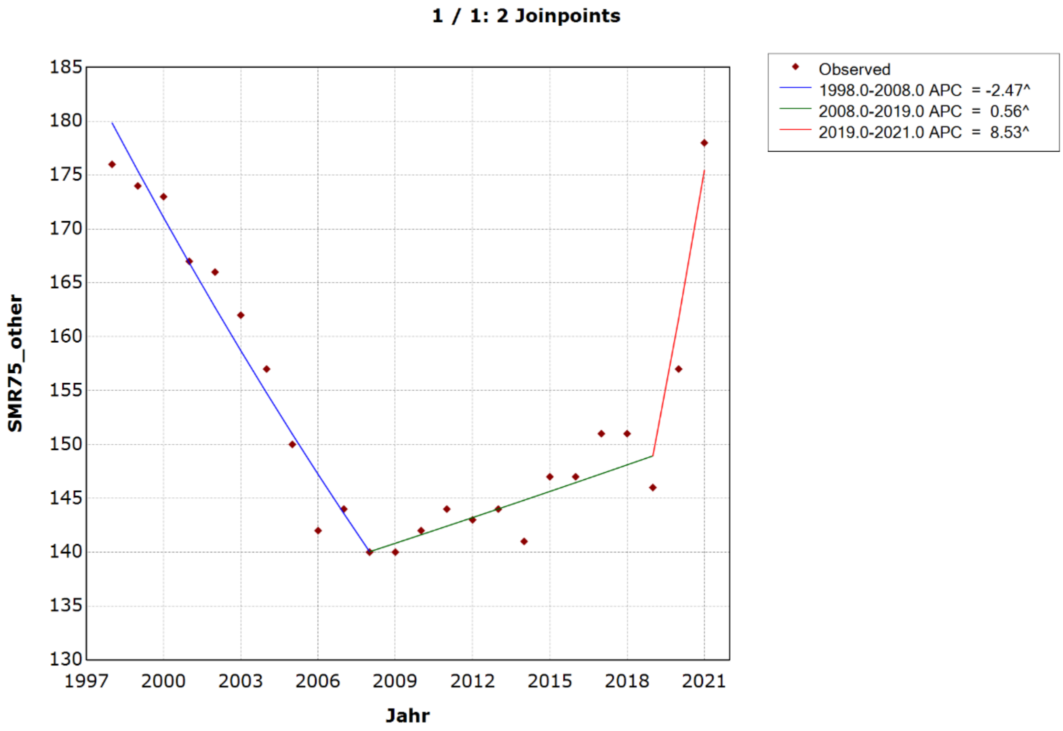

^ Indicates that the Annual Percent Change (APC) is significantly different from zero at the alpha = 0.05 level.
Final Selected Model: 2 Joinpoints.

Männer – hohe Deprivation:

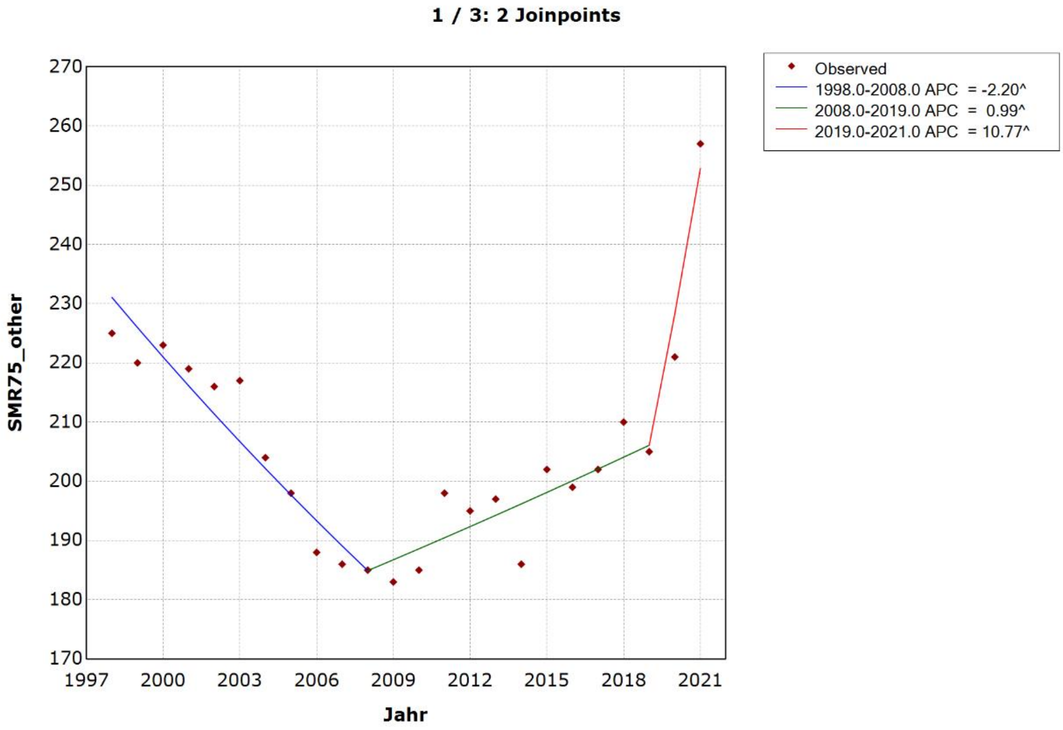

^ Indicates that the Annual Percent Change (APC) is significantly different from zero at the alpha = 0.05 level.
Final Selected Model: 2 Joinpoints.

Todesursachen: andere

Frauen – niedrige Deprivation:

2 / 1: 2 Joinpoints

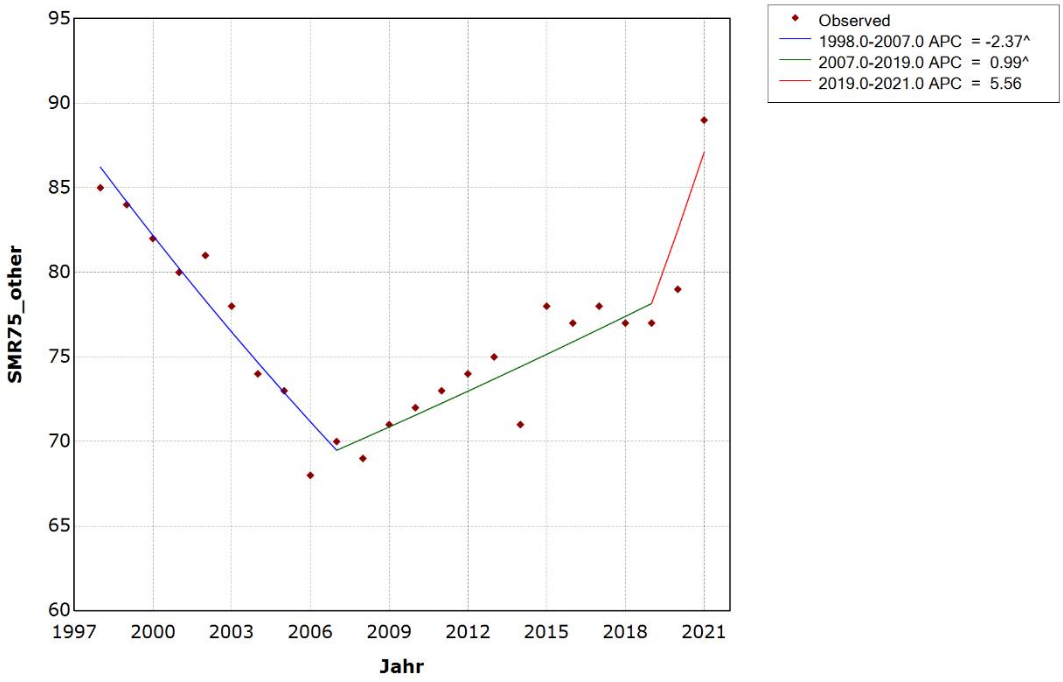

^ Indicates that the Annual Percent Change (APC) is significantly different from zero at the alpha = 0.05 level.
Final Selected Model: 2 Joinpoints.

Frauen – hohe Deprivation:

2 / 3: 2 Joinpoints

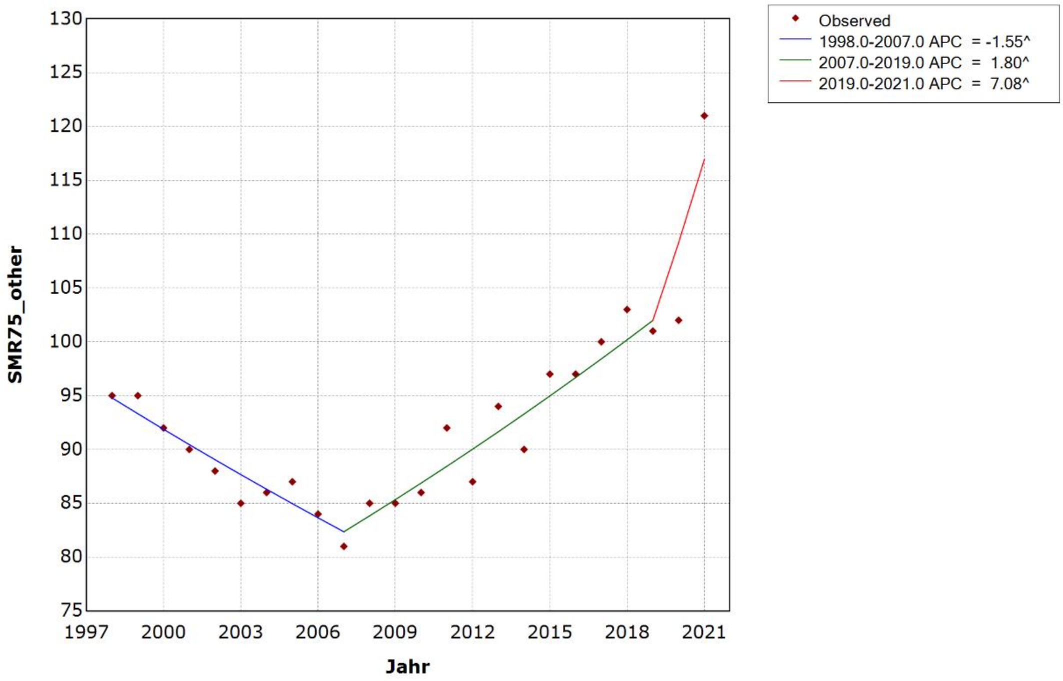

^ Indicates that the Annual Percent Change (APC) is significantly different from zero at the alpha = 0.05 level.
Final Selected Model: 2 Joinpoints.
