# Supplementary material for: Socioeconomic deprivation and premature mortality in Germany, 1998–2021: An ecological study with what-if scenarios of inequality reduction
Source: Bundesgesundheitsblatt Gesundheitsforschung Gesundheitsschutz. 2024 Apr 8;67(5):528–37. [Article in German] doi: 10.1007/s00103-024-03862-0 (PMC11093858; doi:10.1007/s00103-024-03862-0)

Online-Zusatzmaterial 2

Um das Ausmaß absoluter und relativer sozioökonomischer Ungleichheiten in der vorzeitigen Sterblichkeit zu quantifizieren, wurden altersstandardisierte Ratendifferenzen (absolute Ungleichheit) und altersstandardisierte Ratenverhältnisse (relative Ungleichheit) zwischen dem höchsten und niedrigsten Deprivationsquintil stratifiziert nach Geschlecht und Todesursache berechnet. Diese werden auf den folgenden Seiten jahresweise dargestellt und um eine lineare Anpassungsgerade (fitted values) ergänzt, die den durchschnittlichen Zeittrend über den Beobachtungszeitraum anzeigt. Die Ratendifferenz bezieht sich auf den Unterschied in der Anzahl der Sterbefälle pro 100.000 Personen und das Ratenverhältnis auf das Relative Risiko.

Inhaltsübersicht:

- Seite 2: Absolute und relative Ungleichheit (Todesursachen: alle)

- Seite 3: Absolute und relative Ungleichheit (Todesursachen: Herz-Kreislauf-Erkrankungen)

- Seite 4: Absolute und relative Ungleichheit (Todesursachen: Krebserkrankungen)

- Seite 5: Absolute und relative Ungleichheit (Todesursachen: Andere)

Todesursachen: alle

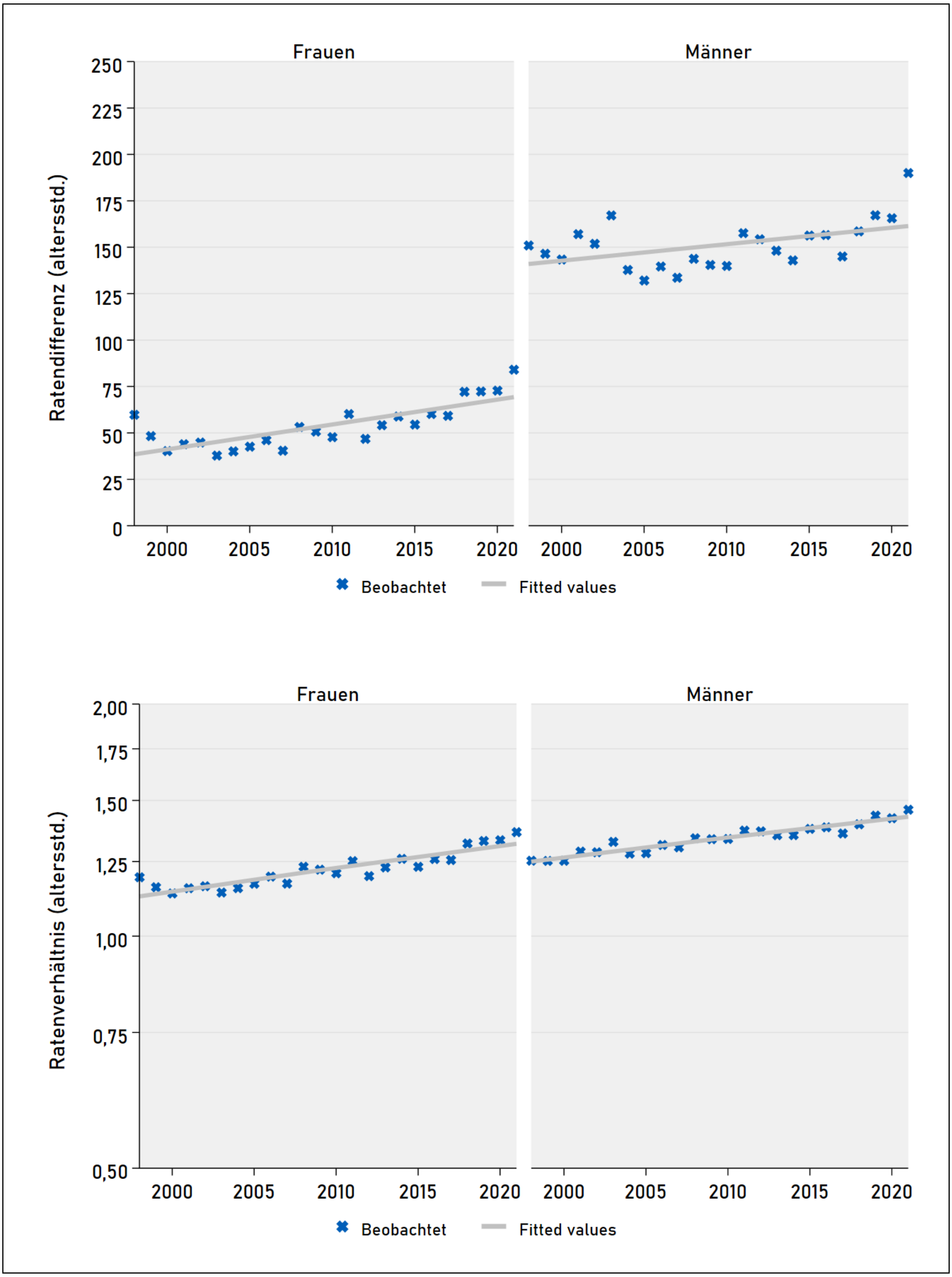

Todesursachen: Herz-Kreislauf-Erkrankungen

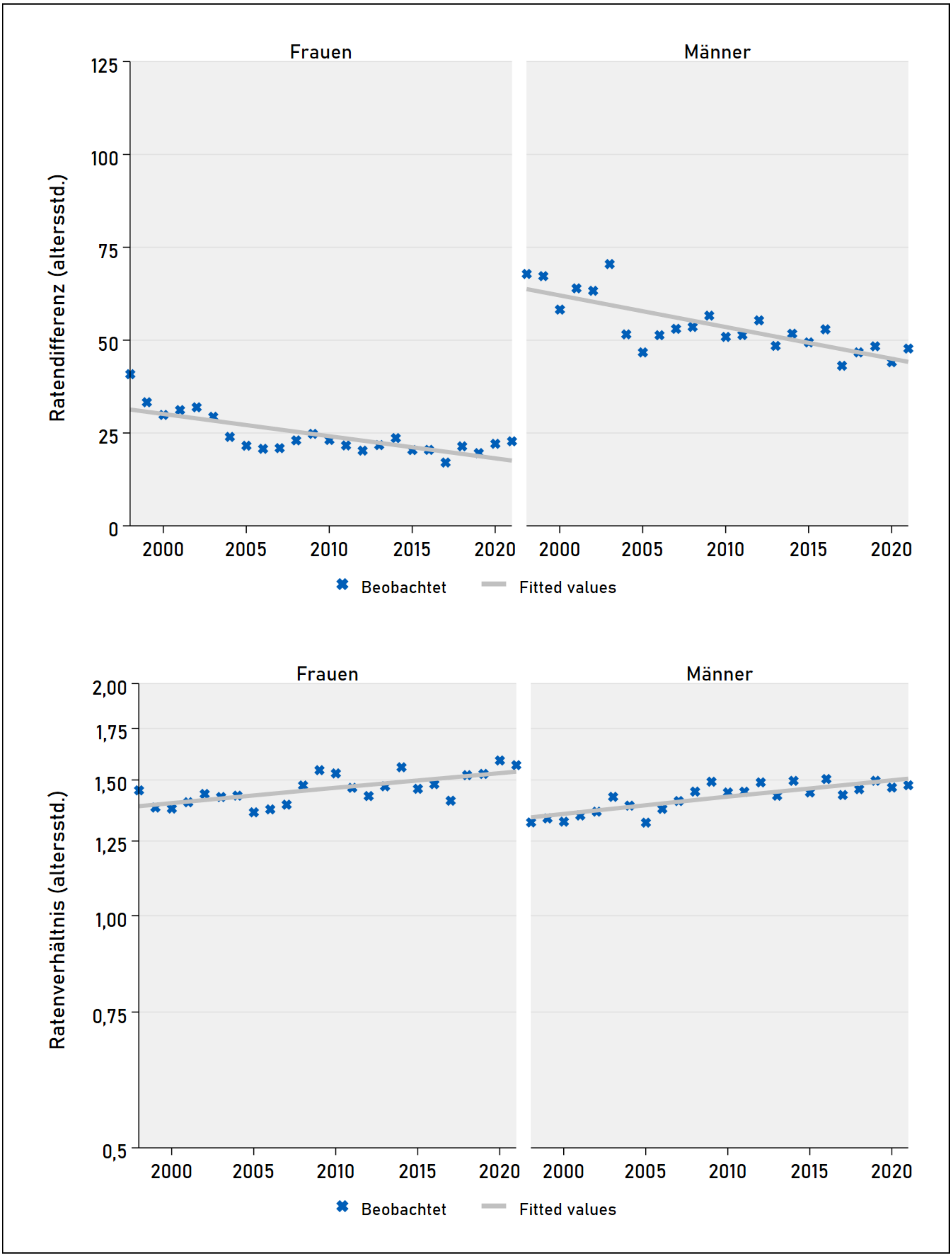

Todesursachen: Krebserkrankungen

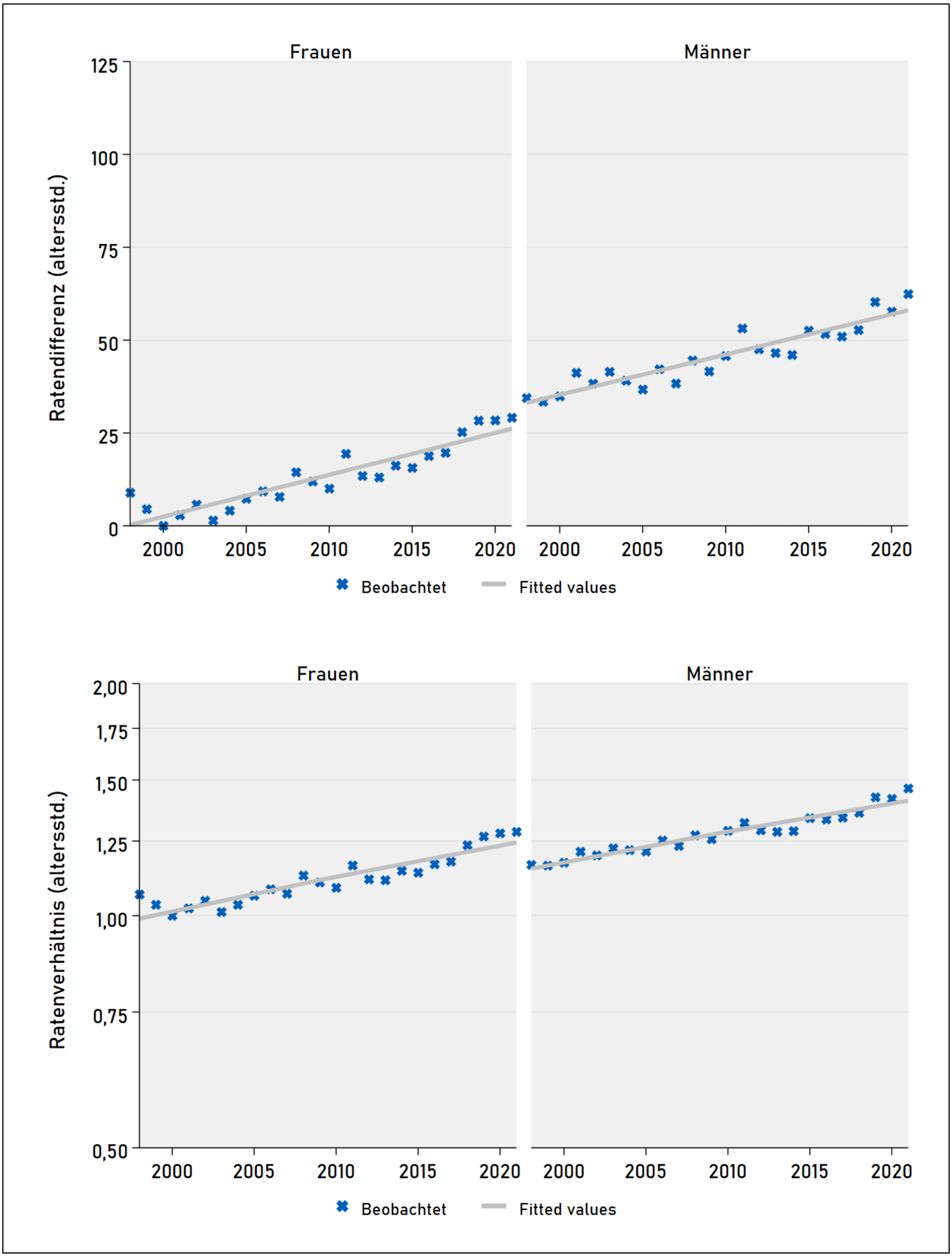

Todesursachen: andere

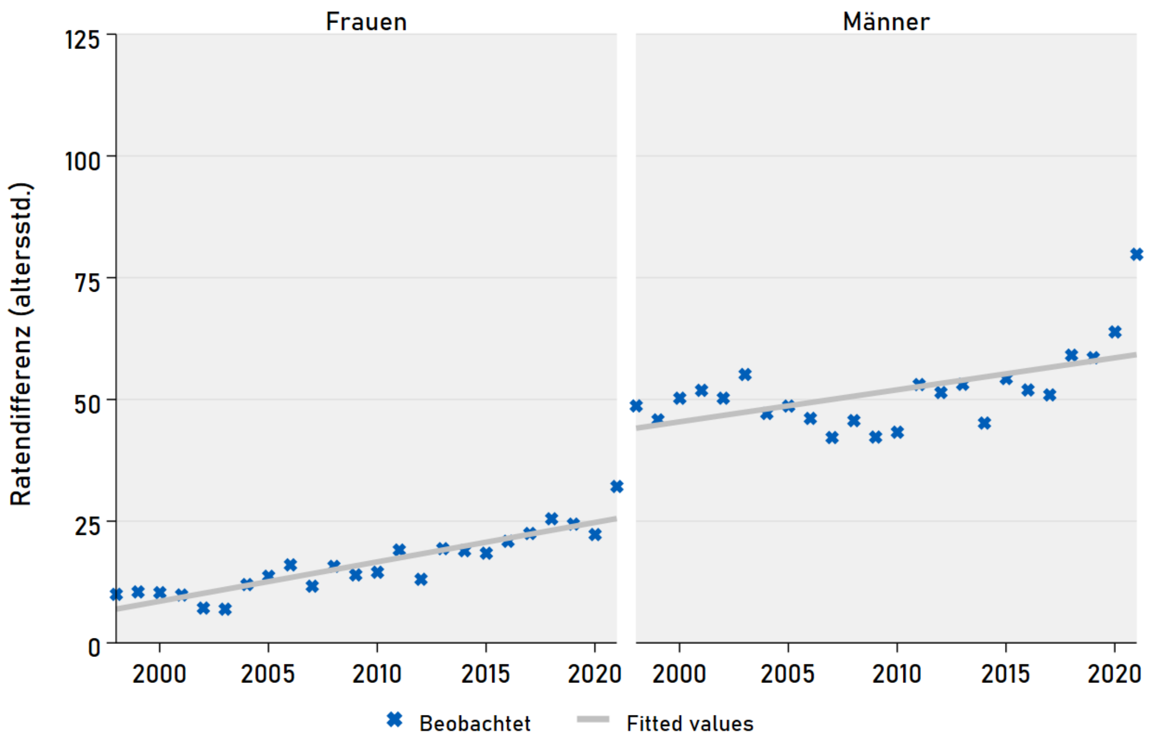

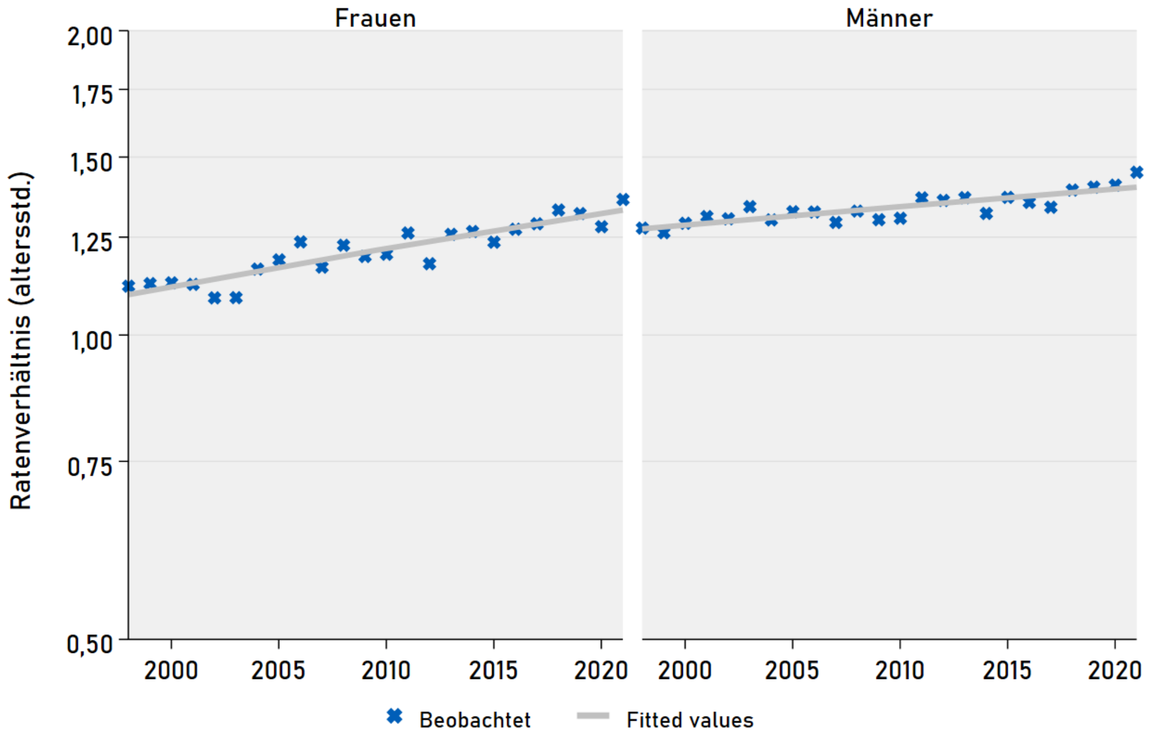

Supplement: Supplementary file 2 [file 103_2024_3862_MOESM2_ESM.pdf]
